# Phase Transitions in the Multi-cellular Regulatory Behavior of Pancreatic Islet Excitability

**DOI:** 10.1371/journal.pcbi.1003819

**Published:** 2014-09-04

**Authors:** Thomas H. Hraha, Matthew J. Westacott, Marina Pozzoli, Aleena M. Notary, P. Mason McClatchey, Richard K. P. Benninger

**Affiliations:** 1 Department of Bioengineering, University of Colorado, Anschutz Medical Campus, Aurora, Colorado, United States of America; 2 Barbara Davis Center for Childhood Diabetes, University of Colorado, Anschutz Medical Campus, Aurora, Colorado, United States of America; McGill University, Canada

## Abstract

The pancreatic islets of Langerhans are multicellular micro-organs integral to maintaining glucose homeostasis through secretion of the hormone insulin. β-cells within the islet exist as a highly coupled electrical network which coordinates electrical activity and insulin release at high glucose, but leads to global suppression at basal glucose. Despite its importance, how network dynamics generate this emergent binary on/off behavior remains to be elucidated. Previous work has suggested that a small threshold of quiescent cells is able to suppress the entire network. By modeling the islet as a Boolean network, we predicted a phase-transition between globally active and inactive states would emerge near this threshold number of cells, indicative of critical behavior. This was tested using islets with an inducible-expression mutation which renders defined numbers of cells electrically inactive, together with pharmacological modulation of electrical activity. This was combined with real-time imaging of intracellular free-calcium activity [Ca^2+^]_i_ and measurement of physiological parameters in mice. As the number of inexcitable cells was increased beyond ∼15%, a phase-transition in islet activity occurred, switching from globally active wild-type behavior to global quiescence. This phase-transition was also seen in insulin secretion and blood glucose, indicating physiological impact. This behavior was reproduced in a multicellular dynamical model suggesting critical behavior in the islet may obey general properties of coupled heterogeneous networks. This study represents the first detailed explanation for how the islet facilitates inhibitory activity in spite of a heterogeneous cell population, as well as the role this plays in diabetes and its reversal. We further explain how islets utilize this critical behavior to leverage cellular heterogeneity and coordinate a robust insulin response with high dynamic range. These findings also give new insight into emergent multicellular dynamics in general which are applicable to many coupled physiological systems, specifically where inhibitory dynamics result from coupled networks.

## Author Summary

As science has successfully broken down the elements of many biological systems, the network dynamics of large-scale cellular interactions has emerged as a new frontier. One way to understand how dynamical elements within large networks behave collectively is via mathematical modeling. Diabetes, which is of increasing international concern, is commonly caused by a deterioration of these complex dynamics in a highly coupled micro-organ called the islet of Langerhans. Therefore, if we are to understand diabetes and how to treat it, we must understand how coupling affects ensemble dynamics. While the role of network connectivity in islet excitation under stimulatory conditions has been well studied, how connectivity also suppresses activity under fasting conditions remains to be elucidated. Here we use two network models of islet connectivity to investigate this process. Using genetically altered islets and pharmacological treatments, we show how suppression of islet activity is solely dependent on a threshold number of inactive cells. We found that the islet exhibits critical behavior in the threshold region, rapidly transitioning from global activity to inactivity. We therefore propose how the islet and multicellular systems in general can generate a robust stimulated response from a heterogeneous cell population.

## Introduction

Most biological systems exist as dynamic multicellular structures where distinct functionalities are generated through cellular interactions. While important for proper function, the complexity in network architecture, cellular dynamics, as well as the presence of heterogeneity, noise and biological variability make the overall function of multicellular structures difficult to understand. Approaches to understanding coupled dynamical systems have handled this complexity by explaining system structure and function individually [1], [2]. These two aspects are both of central importance when it comes to understanding the way living systems are organized and how their anatomy supports their function. Therefore, by employing network theory to inform or predict the architectural aspects of dynamical system models, we can better understand how structural properties can impact functional behaviors. One living system exhibiting complex multicellular dynamics, yet with a scale tractable for study with these approaches, is the islet of Langerhans where dysfunction generally leads to diabetes. As such the islet provides a physiologically relevant system in which we can examine properties of multicellular dynamical systems and discover behavior that is broadly applicable.

The islets of Langerhans are multicellular micro-organs located in the pancreas which maintain glucose homeostasis through the secretion of hormones such as insulin. Glucose-stimulated insulin secretion (GSIS) from β-cells within the islet is driven by glucose-dependent electrical activity. The metabolism of glucose and increased ATP/ADP ratio inhibits ATP-sensitive K^+^ (K_ATP_) channels, causing membrane depolarization. Activation of voltage-dependent Ca^2+^ channels elevates intracellular free-calcium activity ([Ca^2+^]_i_) to trigger insulin granule exocytosis [3], [4]. Defects at several points in this signaling pathway, including the K_ATP_ channel, can cause or enhance the risk of developing diabetes [5]–[8]. Despite the importance of this pathway, it is important to recognize β-cells do not act autonomously. Rather, like many tissues, there are extensive cell-cell interactions within the islet that govern overall function. For example, isolated β-cells exhibit heterogeneous sensitivities to glucose with a low overall dynamic range of GSIS [9]–[11], yet β-cells within the islet robustly release insulin. Connexin36 (Cx36) gap junctions mediate the electrical coupling between β-cells [12]–[14] which coordinates oscillations in electrical activity and insulin release across the islet, enhancing the pulsatile release of insulin and glucose homeostasis [13]–[15]. In the absence of coupling many cells in the islet also show spontaneous elevations in [Ca^2+^]_i_; likely as a result of heterogeneities in glucose sensitivity [10], [16]. Therefore, another equally important role gap junctions play is to coordinate a suppression of spontaneous electrical activity at lower glucose levels [17]. Given that basal regulation is integral to glucose homeostasis, electrical coupling and the coordinated electrical dynamics are a critical factor in the regulation of islet function and in diabetes.

Multicellular electrical dynamics in the islet have been described as functional networks where synchronized changes in [Ca^2+^]_i_ indicate functional connectivity between cells [14], [18], [19]. Such network analysis has been applied to examine the dependence of [Ca^2+^]_i_ dynamics on the level of coupling and its regulation, and has indicated that β-cell connectivity is non-homogeneous with a small subset of connections dominating synchronized behavior. As part of this analysis, the network of functional connectivity can be approximated by a Boolean network which quantitatively describes changes in multicellular behavior, including changes in coupling strength, network size or network shape [14], [20]–[22]. These studies have generally focused on the synchronization of [Ca^2+^]_i_ oscillations, and such synchronized oscillatory/pulsatile behavior has been similarly examined in other physiological multicellular systems [23]–[25]. However, few studies have theoretically examined the suppressive effect of electrical coupling in the islet and its ability to shape the glucose-regulation of electrical activity. This is particularly warranted given a recent study that showed how severe diabetes caused by expression of mutant K_ATP_ channels could be prevented through a modulation in gap junction coupling [26]. Therefore, details for how the network structure and composition facilitate a highly sensitive and robust response from a heterogeneous cell population remain to be determined.

In this study we examine how electrical coupling within β-cell networks in the islet provide resilience against heterogeneous cell populations to generate robust network responses. We first develop quantitative predictions derived from a Boolean approximation of the β-cell network, where the dependence of [Ca^2+^]_i_ on variations in the constituent cellular excitability and coupling is described. We then test these predictions using two experimental systems involving transgenic mice that express mutant K_ATP_ channels with increased or decreased ATP-sensitivity [27], [28]. This creates defined populations of cells within the islet which are ‘excitable’ or ‘inexcitable’, and can be further used to examine how our theoretical predictions and experimental data extend to physiological regulation of glucose homeostasis. We next link the static Boolean network model predictions and experimental findings with a dynamic multicellular model of the islet which incorporates recent understanding of β-cell electrophysiology [29], [30]. We finally extend these experimental and theoretical measurements to a general case with a continuum of heterogeneous cellular behavior.

A consistent feature in this study is the emergence of critical behavior as a result of β-cell electrical coupling, where the islet exhibits a phase transition between globally active and inactive states as cellular excitability approaches a critical threshold value. We discuss how the robust functionality that emerges at the multicellular level is not only relevant to the islet of Langerhans and its dysfunction in diabetes, but also to the function of other multicellular biological systems.

## Results

### Boolean Network Model Predicts Phase Transition in [Ca^2+^]_i_

**Figure 1:**
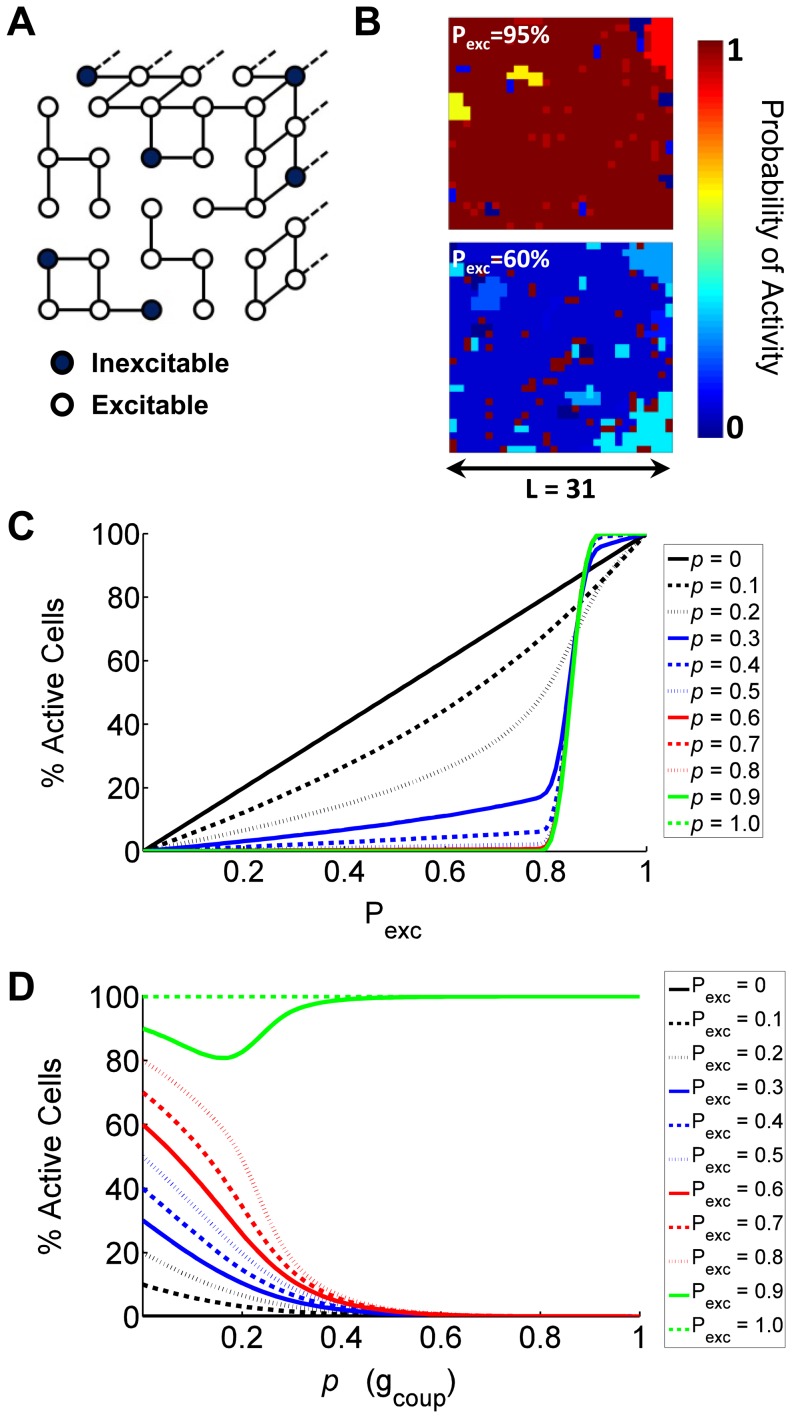
Boolean network model predictions. A) Schematic representation of the network model with limited connectivity. Note larger connected clusters have a higher probability of containing inexcitable cells. B) Example false-color maps displaying probability of activity, generated from a simulated network with *p* = 0.30 at P_exc_ = 95% (top) and P_exc_ = 60% (bottom). Note substantially increased likelihood of activity with the higher P_exc_. Further description for how this was generated can be found in figure S1. C) Boolean network model predictions for the mean percent active cells as a function of proportion of excitable cells (P_exc_) for varying coupling probabilities *p*, with a threshold fraction of inexcitable cells *Sp* = 0.15. D) Boolean network model predictions for the mean percent active cells as a function of coupling probability *p*, for varying proportion of excitable cells P_exc_, where *Sp* = 0.15.

Based on prior approximations of heterogeneity in cellular excitability and coupling, Boolean networks of connectivity were simulated to predict how general multicellular electrical activity depends on the relative excitability of the constituent cell population and the coupling between individual cells [14], [20]. Nodes within a cubic lattice had a probability P_exc_ of being active, and adjacent nodes were functionally coupled with a ‘coupling probability’ *p* (figure 1A), and resultant clusters of coupled nodes were identified. ‘Inexcitable’ β-cells can suppress activity in excitable β-cells via electrical coupling [17], with <30% inexcitable cells necessary for this suppression. To simulate this, a logic rule was used for each cluster of coupled nodes within a given lattice, where greater than a threshold percentage of inexcitable cells (*Sp*) can suppress activity in all other cells in its coupled network. Simulations of the resultant average network activity were run with varying values of P_exc_, *Sp*, and *p* to represent differing cellular excitabilities and electrical coupling (figure 1B, SI figure S1).

An increase in electrical activity is predicted as P_exc_ is increased; however the functional form is highly dependent on *p* (figure 1C). In the absence of coupling (*p* = 0), a trivial linear response is obtained where P_exc_ represents the level of electrical activity. With increasing *p*, the activity becomes increasingly non-linear as a function of P_exc_. For higher values of *p* (0.3 to 1) a sharp transition between active and quiescent behavior is observed, representing a phase transition with emerging critical behavior. These higher values of *p* lead to network-spanning coupling (figure S1, S2), and as such the ‘rule’ governing suppression acts over the whole network. For low values of *p* (0 to 0.2), the network is composed of coupled ‘clusters’ (figure S2), and the simulation is close to linear without a strong transition. This level of *p* corresponds to insufficient coupling to span the network, which is similar to the critical coupling probability (∼0.25) in percolation theory [31]. As such for *p*>0.25 there are 3 specific regions of emergent network behavior: a small (∼10%) decrease for P_exc_>0.85 (‘pre-critical state’); a rapid ∼75% drop at P_exc_ = 0.85 (‘critical state’), then a small linear decrease for P_exc_<0.85 (‘post-critical state’). The critical state emerges when P_exc_ approaches 1-*Sp*; with the sharpness of the transition as well as behavior in the pre- and post-critical states being dependent on *p*.

Overall, this transition can be understood by considering the well-defined threshold for activity (*Sp*) and the network-spanning connectivity that occurs above the critical coupling probability (*p*>0.25). For values of P_exc_<(1-*Sp*) there is a gradual decrease in network activity with increasing *p* (figure 1D), representing the suppressive effect of coupling. For P_exc_>(1-*Sp*), the network activity remains high, although a slight drop occurs for low levels of coupling. Therefore in a general Boolean network, electrical coupling is predicted to lead to critical behavior, where a phase transition in the network activity occurs as a function of constituent cellular activity.

### Phase Transition in [Ca^2+^]_i_ in Kir6.2^[ΔN30,K185Q]^ Expressing Islets

To test the Boolean network model predictions, we measured intracellular free-calcium activity ([Ca^2+^]_i_) in islets which had defined levels of cellular excitability. Islets were isolated from mice with inducible, β-cell specific expression of a mutant ATP-insensitive K_ATP_ channel subunit (Kir6.2^[ΔN30,K185Q]^) under Cre^ER^-recombinase control [27]. Expression of these over-active K_ATP_ channels render β-cells functionally inexcitable, causing an absence of insulin release, marked hyperglycemia and diabetes [27]. Tamoxifen induction of Cre^ER^ controls Kir6.2^[ΔN30,K185Q]^ expression levels which can be monitored via GFP co-expression, leading to both controllable and quantifiable cellular excitabilities (SI figure S3).

**Figure 2:**
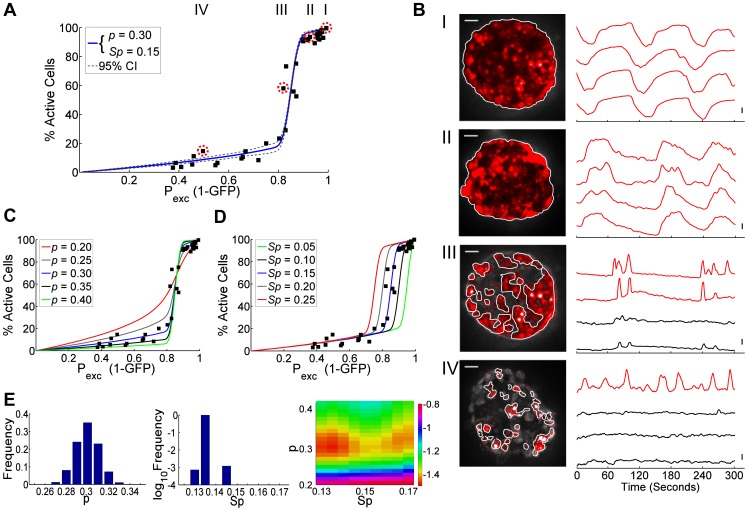
Experimental data showing how Boolean network model describes phase transitions in islet [Ca^2**+**^]_i_. A) Percent cells showing [Ca^2+^]_i_ elevations as a function of number of excitable cells, as determined by lack of GFP and thus Kir6.2^[ΔN30,K185Q]^ expression (i.e. P_exc_ = 1-%GFP), together with Boolean network model fit. Filled squares indicate experimental data, solid line represents mean of simulations that best fit data with *p* = 0.30 and *Sp* = 0.15 (χ^2^ = 1.38), dashed lines represents 95% confidence intervals of the simulation fit. B) Representative [Ca^2+^]_i_ data for islets indicated in A, from regions of wild-type (I), ‘pre-critical’ (II), ‘critical’ (III) and ‘post-critical’ (IV) levels of P_exc_. Left: Areas of activity are highlighted in red and scale bars represent 50 µm. Right: Representative time-courses of normalized FuraRed calcium dye fluorescence for cells within each islet, where vertical scale bar indicates 20% change in fluorescence. Red time-courses are determined to be active, black time-courses are determined to be inactive. See SI for Movies S1, S2, S3, S4 of these data. C) Experimental data with Boolean network simulations for varying connectivity *p*. D) As in C for varying threshold of inactive cells *Sp*. E) Probability distribution of fitted *p* (linear scale) and *Sp* (log scale) parameters to data in A, along with heat map of 2D χ^2^ distribution (log scale).

At 20 mM glucose islets show [Ca^2+^]_i_ which decreased with increasing expression of GFP and therefore Kir6.2^[ΔN30,K185Q]^, similar to model predictions. This showed critical behavior with 3 specific regions (figure 2A): For low GFP expression <15% (few Kir6.2^[ΔN30,K185Q]^ expressing cells), [Ca^2+^]_i_ was active over the entire islet with similar behavior to wild-type islets lacking GFP (GFP = 0) and Kir6.2^[ΔN30,K185Q]^ (figure 2A, BI–II, ‘pre-critical’ behavior). Oscillations were almost fully synchronous in each case (not shown). For GFP expression at 10–20% there was a sharp drop-off in islet [Ca^2+^]_i_, where small changes in GFP resulted in highly disproportionate changes in [Ca^2+^]_i_ (figure 2A, BIII, ‘critical’ behavior). Activity was focused to clustered areas of synchronization (not shown). For high GFP expression >25% (high number of Kir6.2^[ΔN30,K185Q]^ expressing cells), islets showed sporadic [Ca^2+^]_i_ restricted to increasingly smaller clusters (Figure 2DIV, ‘post-critical behavior’). Although islets with <20% GFP has similar overall activity compared to wild-type islets (0% GFP), there was a marked reduction in the plateau fraction in the <20% GFP group (15–40%) compared to the wild-type group (50–70%); indicating that even small numbers of inactive cells impacts global behavior. In comparison, islets with high levels of GFP (>25%) had a low plateau fraction (15–20%) in those cells that were active. GFP^+^ cells showed similar activity to GFP^−^ cells albeit with a small increase in activity, likely due to a few inactive non-β-cells included in the GFP^−^ analysis (SI figure S4).

Comparison of experimental data to the Boolean model can be seen for a number of values of *p* (figure 2C) and *Sp* (figure 2D). Varying *p* (gap junction coupling) matches the sharpness of the transition, whereas varying *Sp* (number of inexcitable cells required to suppress activity) matches the position of the transition. A *p* = 0.3 (95% CI: 0.280–0.311) and *Sp* = 0.135–0.15 best fits the experimental [Ca^2+^]_i_ data (figure 2A,E). The distribution for fitted *p* was relatively broad but for *Sp* was well defined (figure 2E). These values of *p* are similar to those found in prior studies examining the synchronization of [Ca^2+^]_i_ oscillations, which indicated a limited level of functional coupling in the islet (*p* = 0.31–0.36) [20]. These values of *Sp* are also consistent with experimental studies that suggest between 1 and 30% of inactive cells can suppress activity in other cells [17], [20].

Therefore, introducing inexcitable cells into the islet experimentally generates critical behavior which quantitatively agrees with a Boolean network model and predicts the importance of electrical coupling in regulating multicellular excitability.

### Phase Transition in Physiological Parameters

**Figure 3:**
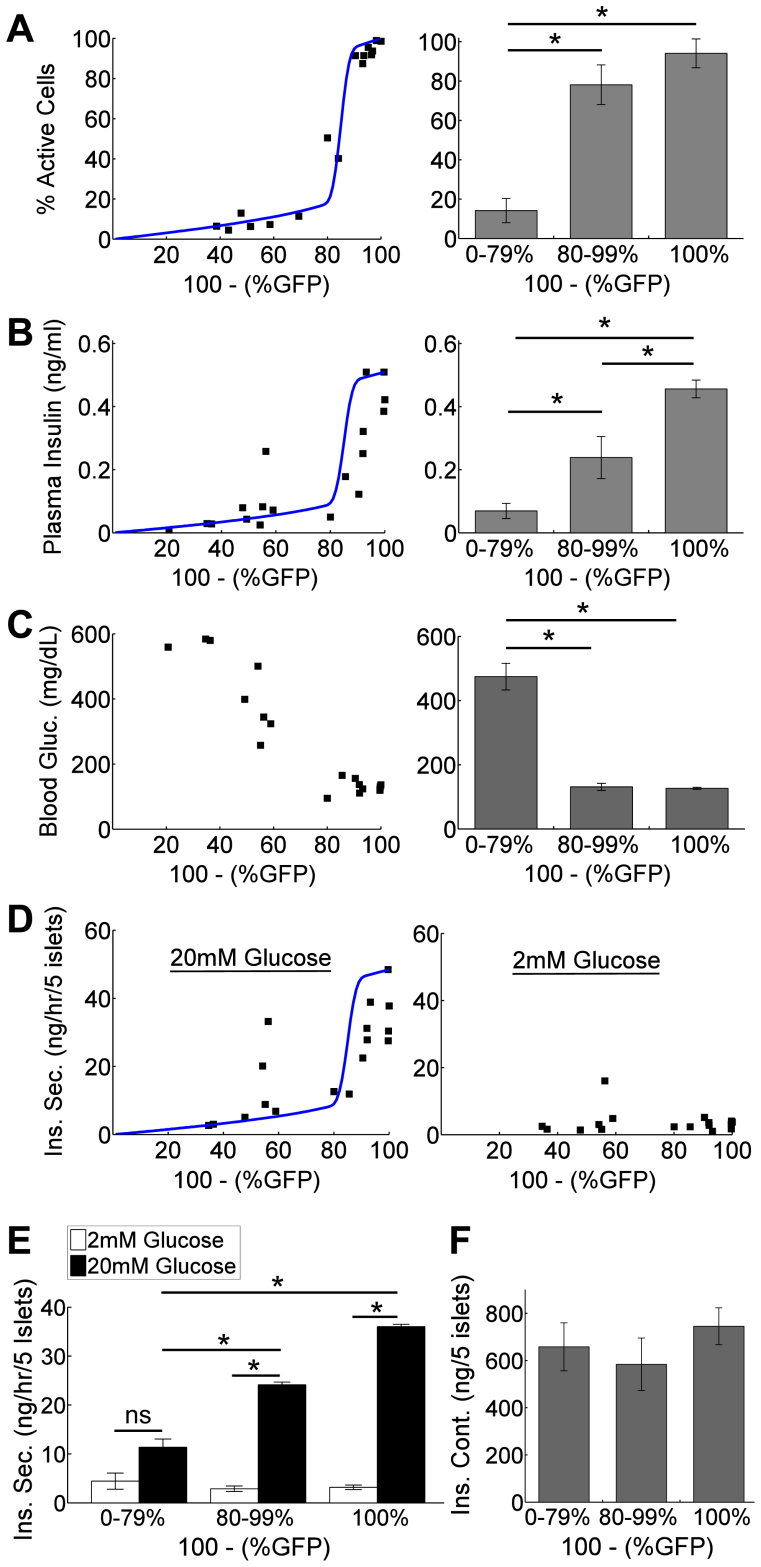
Link between phase transitions in [Ca^2**+**^]_i_ and physiological parameters. A) Percent cells showing [Ca^2+^]_i_ elevations averaged over islets from each Kir6.2^[ΔN30,K185Q]^-expressing mouse as a function of P_exc_ (100%-%GFP). Right: Mean(±s.e.m.) for data binned to wild-type, pre- and post-critical ranges as determined by %GFP. *indicates significant difference (p<0.0001) between data as indicated. B) Plasma insulin levels from each mouse as a function of P_exc_. Right: Mean(±s.e.m.) for data binned as in A. *indicates significant difference (p<0.05) between data as indicated. C) Time-averaged blood glucose levels from each mouse as a function of P_exc_. Right: Mean(±s.e.m.) for data binned as in A. *indicates significant difference (p<0.0001) between data as indicated. D) Insulin secretion from isolated islets at 20 mM glucose (left) and 2 mM glucose (right), averaged over each mouse as a function of P_exc_. E) Mean(±s.e.m.) for data in D binned as in A. *indicates significant difference (p<0.01) between data, ‘ns’ indicates no significant difference (p>0.05) as indicated. F) Mean(±s.e.m.) of islet insulin content averaged over each mouse. Solid lines in A,B,D represent Boolean model fit (*p* = 0.3) from experimental data in Figure 2C.

β-cell [Ca^2+^]_i_ drives insulin release to regulate glucose homeostasis. Given that the behavior in [Ca^2+^]_i_ following varied expression of over-active K_ATP_ channels, we next tested whether this also occurred in downstream physiological parameters. Averaged over each mouse, similar [Ca^2+^]_i_ was observed in wild-type islets lacking GFP and islets with low-level GFP (<20%, ‘pre-critical’), while both were significantly greater than [Ca^2+^]_i_ in islets with high GFP expression (>20%, ‘post-critical’) (figure 3A). Plasma insulin also showed a similar transition, with pre-critical (GFP<20%) plasma insulin being significantly greater than post-critical (GFP>20%) plasma insulin (figure 3B). However mice lacking GFP did show significantly greater insulin than mice with low-level GFP, correlating with the reduced plateau fraction observed. Insulin reduces glucose levels, and as expected pre-critical mice (GFP<20%) had normal glucose levels (figure 3C), while post-critical mice (GFP>20%) demonstrated elevated glucose levels. Glucose-stimulated insulin secretion from isolated islets showed similar behavior to that of plasma insulin (figures 3D–F), where again islets lacking GFP showed significantly greater GSIS than islets with low level GFP.

Therefore insulin dynamics and blood glucose levels follow similar behavior as the driving [Ca^2+^]_i_ following varied expression of Kir6.2^[ΔN30,K185Q]^, demonstrating a physiological link in the critical behavior in [Ca^2+^]_i_ activity as a function of P_exc,_.

### Coupling Dependent Suppression in Kir6.2^[AAA]^ Expressing Islets

**Figure 4:**
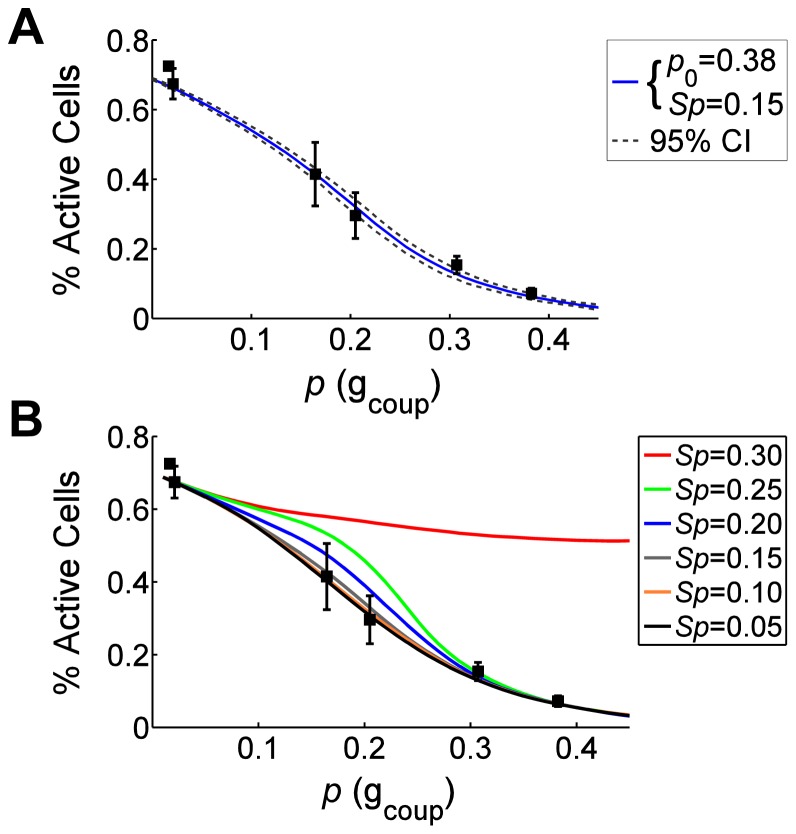
Boolean network model describes [Ca^2**+**^]_i_ suppression as a function of coupling conductance. A) Percent cells showing [Ca^2+^]_i_ elevations in islets from Kir6.2^[AAA]^-expressing mice as a function of gap junction conductance, together with Boolean network model fit. Filled squares indicate mean(±s.e.m.) experimental data, solid line represents mean of simulations that best fit data for wild-type coupling value *p_0_* = 0.38 and *Sp* = 0.15 (χ^2^ = 0.416), dashed lines represents 95% confidence intervals of simulation fit. Gap junction conductance for each data point was normalized to the wild-type conductance and scaled by the fitted *p_0_*. B) Mean(±s.e.m.) experimental data with Boolean network simulations for varying threshold of inactive cells *Sp*.

The Boolean model accurately predicts the impact of variable cellular excitabilities (P_exc_) on [Ca^2+^]_i_ suppression at elevated glucose through expression of over-active K_ATP_ channels (Kir6.2^[ΔN30,K185Q]^). However, the Boolean model also predicts how [Ca^2+^]_i_ suppression varies as a function of gap junction coupling (*p*) (Figure 1D). To test this, we measured [Ca^2+^]_i_ in islets from mice with β-cell specific mosaic expression of an inactive K_ATP_ channel subunit (Kir6.2^[AAA]^). This was combined with a knockout of Cx36, yielding 100% (Cx36^+/+^), 50% (Cx36^+/−^) or 0% (Cx36^−/−^) gap junction coupling, as well application of the gap junction inhibitor 18-α-glycyrrhetinic acid [10]. Expression of inactive K_ATP_ channels render β-cells constitutively (glucose-independent) active, yet islets which have a majority (but not all) of their cells expressing inactive K_ATP_ channels show glucose-dependent electrical activity similar to wild-type islets [17]. GFP co-expression indicates ∼70% of β-cells express inactive K_ATP_ channels (SI figure S3) such that P_exc_ = 0.7. With increasing gap junction coupling [Ca^2+^]_i_ progressively decreased until residual activity was observed at full coupling, similar to that in the post-critical state upon Kir6.2^[ΔN30,K185Q]^ expression. There was strong agreement between experimental measurements and the Boolean Network model, with a *p* at normal (Cx36^+/+^, 100%) gap junction coupling of 0.38 (95% CI: 0.372–0.394) and a suppression threshold *Sp* = 0.15 giving the best fit (figure 4A). This is similar to *p*, *Sp* derived in the first experimental system (figure 2). Varying *Sp* affects the gap junction dependence in [Ca^2+^]_i_, with little effect between 0.05–0.2, but strong divergence above 0.2 (figure 4B). Therefore the Boolean network model can accurately predict behavior in a different experimental model with defined levels of cellular excitability (P_exc_) and gap junction coupling (*p*).

### Phase Transition in a Dynamical Model of β-Cell Networks

The Boolean network model accurately describes how [Ca^2+^]_i_ critically depends on cellular excitability and coupling. Nevertheless it is a static framework of a dynamical system and does not take into account limit-cycle behavior. To investigate whether similar behavior exists in a coupled dynamical oscillator model of the islet, we generated a multi-cellular version of a recent β-cell model which includes a comprehensive description of β-cell electrophysiology [30]. Our model also included a more realistic quasi-spherical architecture, heterogeneity in gap junction coupling [14], [32], and heterogeneity in endogenous cellular activity [11], [14]. To model K_ATP_-overactivity resulting from Kir6.2^[ΔN30,K185Q]^ expression, a defined fraction of cells with reduced ATP-inhibition of K_ATP_ activity was introduced to render them inexcitable.

**Figure 5:**
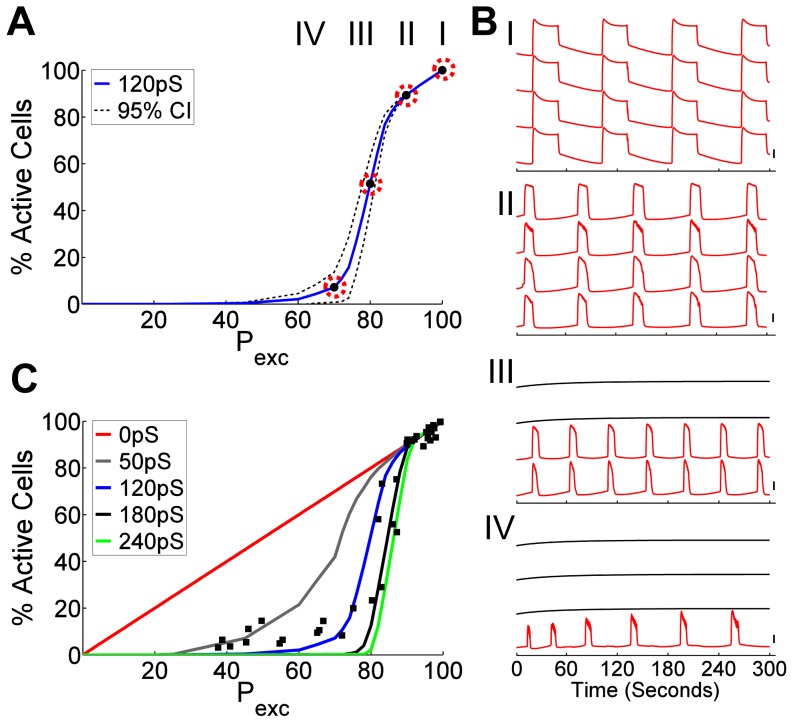
Coupled dynamical oscillator model describes experimental islet phase transitions. A) Percent cells showing [Ca^2+^]_i_ elevations in simulated islets as a function of fraction of excitable cells (P_exc_), as set by the % cells lacking ATP-insensitivity. Solid line represents mean of simulation results generated from 5 random number seeds, dashed lines represents 95% confidence intervals of simulations. B) Representative simulated [Ca^2+^]_i_ time-courses for parameters indicated in A, from regions of wild-type (I), ‘pre-critical’ (II), ‘critical’ (III) and ‘post-critical’ (IV) behavior, as in figure 2. Vertical scale bar indicates 20% change in simulated [Ca^2+^]_i_. Red time-courses are determined to be active, black time-courses are determined to be inactive. See SI for Movies S5, S6, S7, S8 of these data. C) Percent cells showing [Ca^2+^]_i_ elevations in simulated islet as a function of number of excitable cells (P_exc_) for varying mean gap junction conductance values. Filled squares indicate experimental data from Kir6.2^[ΔN30,K185Q]^-expressing islets in figure 2.

As with the Boolean network model and experimentally measured [Ca^2+^]_i_, a clear phase transition was observed at 20 mM glucose in the coupled oscillator model with ∼15% K_ATP_-overactivity (figure 5A). Again critical behavior manifested in three regimes. Simulated islets without K_ATP_ over-activity showed [Ca^2+^]_i_ dynamics closely matching previously published models (figure 5BI) [30]. Simulated islets with low K_ATP_-overactivity (<15%) showed a linear decrease in activity with a reduced plateau fraction as experimentally observed (figure 5A, BII, ‘pre-critical’ behavior), while maintaining near-full synchronization. Simulated islets with K_ATP_-overactivity at 10–30% again showed a sharp drop-off in [Ca^2+^]_i_, with small changes in K_ATP_-overactivity leading to highly disproportionate changes in [Ca^2+^]_i_ (figure 5A, BIII). Simulated islets with high K_ATP_-overactivity (>30%) showed only sporadic low level [Ca^2+^]_i_ (Figure 5A, BIV, ‘post-critical’ behavior). A physiological mean gap junction conductance of 120 pS [14], [32] was found to best describe experimental data (figure 5A,C). The sharpness and position of the phase transition was highly dependent on the mean coupling conductance, with increasing conductance leading to a sharper transition occurring at lower K_ATP_-overactivity (figure 5C).

The islet is commonly modeled as a cubic lattice or other regular geometry [14], [21], [33]. A spherical islet-like structure which has a heterogeneous number of cell-cell connections (mean,SD = 5.3,1.7) generated a less-sharp transition compared to a regular cubic geometry, and this better matched experimental data (SI figure S5). Similarly, a heterogeneous level of coupling conductance generated a less-sharp transition (SI figure S6A). This indicates the importance of coupling heterogeneity, in terms of connection geometry, connection number and connection strength. The endogenous heterogeneity in cellular activity did not significantly impact the phase-transition indicating the dominating effect of Kir6.2^[ΔN30,K185Q]^ expression (SI figure S6B). A similar phase-transition was also observed for simulations run at 11 mM glucose (not shown).

Therefore critical behavior also emerges in a dynamic coupled β-cell oscillator model with quantitative agreement with experimental measurements and a static Boolean network model.

### Phase Transition Resulting from Endogenous Heterogeneity and Coupling

We have examined how the coupling between heterogeneous cells leads to critical behavior by introducing defined mutant populations of inexcitable cells (Kir6.2^[ΔN30,K185Q]^) or excitable cells (Kir6.2^[AAA]^). However, endogenous β-cells are themselves highly heterogeneous under physiological ranges of glucose, showing a continuum of excitabilities rather than being constitutively excitable/inexcitable [9]–[11].

**Figure 6:**
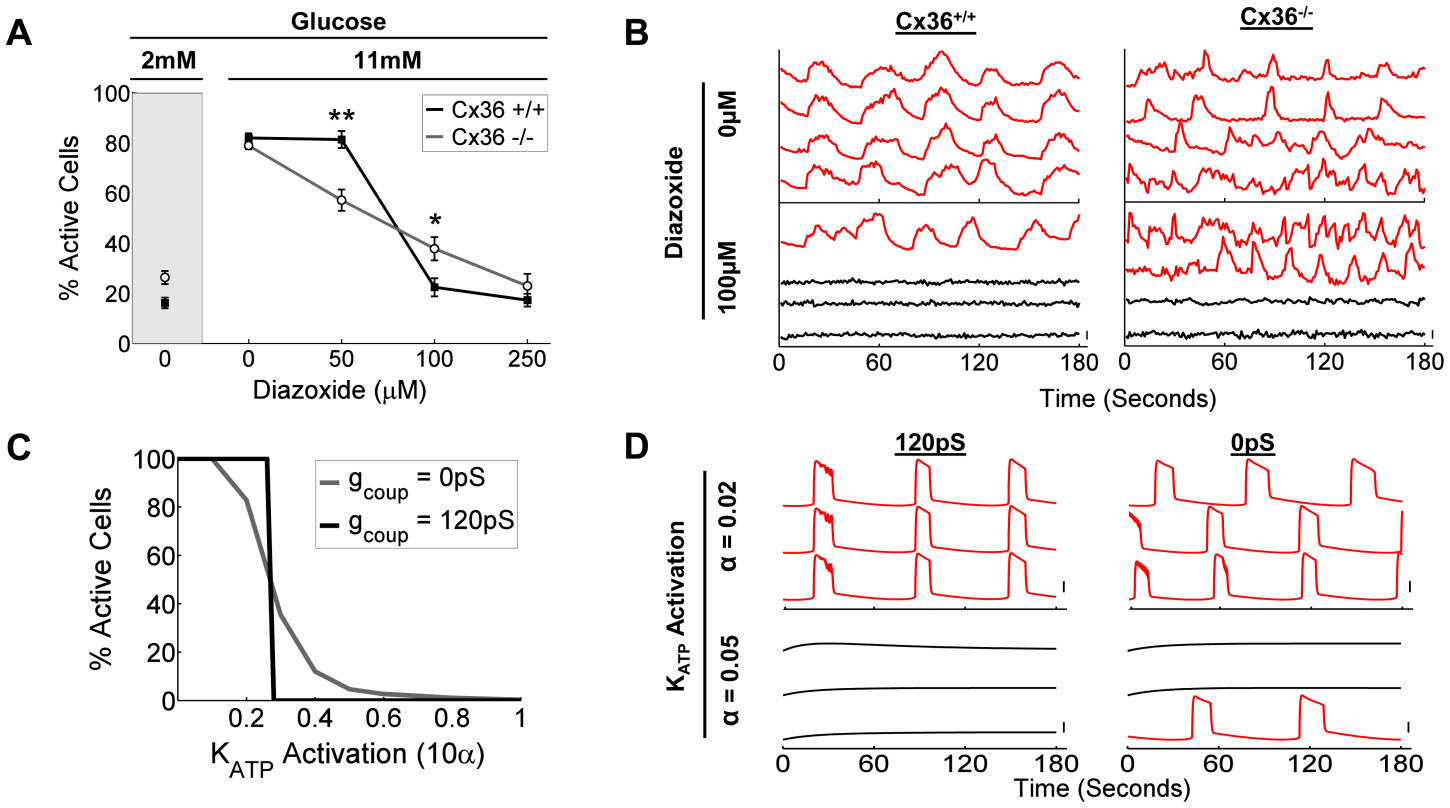
Coupling-dependent phase transitions upon uniform K_ATP_ inhibition resulting from endogenous heterogeneity. A) Mean(±s.e.m.) percent cells showing [Ca^2+^]_i_ elevations at 2 mM glucose and at 11 mM glucose with 0, 50, 100, 250 µM of the K_ATP_ activator diazoxide, for wild type islets (Cx36^+/+^, solid squares) and islets lacking gap junction coupling (Cx36^−/−^, empty circles). *, ** indicate significant difference (p<0.05, p<0.005) between activity in Cx36^+/+^ and Cx36^−/−^ islets at treatments indicated. B) Representative [Ca^2+^]_i_ time-courses for Cx36^+/+^ and Cx36^−/−^ islets at 11 mM glucose with 0 µM or 100 µM diazoxide. Red time-courses are determined to be active, black time-courses are determined to be inactive. Vertical scale bar indicates 20% change in fluorescence. C) Percent cells showing [Ca^2+^]_i_ elevations in simulated islet as a function of a uniform increase in the fraction of ATP-insensitivity of K_ATP_ channel activation (α) across cells of the islet. Mean simulation data is presented for zero gap junction conductance (0 pS) and wild-type gap junction conductance (120 pS). D) Representative simulated [Ca^2+^]_i_ time-courses for wild-type gap junction conductance (120 pS) and zero gap junction conductance upon indicated levels of uniform K_ATP_ activation. Red time-courses are determined to be active, black time-courses are determined to be inactive. Vertical scale bar indicates 20% change in simulated [Ca^2+^]_i_.

To examine how gap junction coupling leads to critical behavior in the presence of endogenous heterogeneity, we applied a ‘ramp’ of increasing diazoxide concentrations to uniformly promote K_ATP_ channel opening. At 11 mM glucose, [Ca^2+^]_i_ in wild-type islets at 0 µM and 50 µM diazoxide was similar, but at 100 µM there was a rapid ∼60% drop (figure 6A), where only a few remaining cells were active (figure 6B). Similar low-level [Ca^2+^]_i_ was observed at 250 µM. In islets from mice lacking Cx36 gap junction coupling, similar [Ca^2+^]_i_ was observed to wild-type islets at 0 µM diazoxide, albeit with no synchronization. Upon increasing diazoxide, a more gradual decrease in [Ca^2+^]_i_ was observed, with less [Ca^2+^]_i_ observed at 50 µM diaozixde but more [Ca^2+^]_i_ remained at 100 µM diazoxide (figure 6A,B).

These data were also well described using the coupled dynamic oscillator model. In the presence of endogenous heterogeneity at 11 mM glucose, a uniform reduction in ATP-sensitive K_ATP_ inhibition led to a clear phase transition in islet [Ca^2+^]_i_ in the presence of normal coupling (120 pS) (figure 6C,D). However, in the absence of coupling a more gradual change occurred in good agreement with experimental measurements (figure 6C,D); where [Ca^2+^]_i_ was elevated in the absence of coupling over a certain range of uniform K_ATP_ inhibition. As such, <50 µM diazoxide lies in the ‘pre-critical’ regime, >100 µM diazoxide lies in the ‘post-critical’ regime, and the transition lies at 50–100 µM.

**Figure 7:**
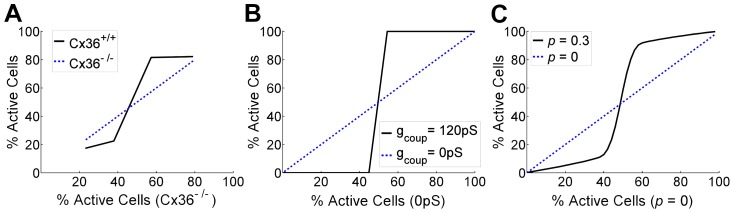
Phase transitions in endogenous β-cell network activity, as shown by the activity in a fully-coupled islet system as a function of the activity in the uncoupled islet system; where the latter represents the intrinsic excitability of the constituent cells. A) Experimentally measured transition from global activity to quiescence in wild-type islets treated with varying diazoxide concentrations, showing phase transition in activity as constituent cellular activity is reduced B) Simulated transition from global activity to quiescence upon normal gap junction conductance as K_ATP_ is uniformly activated across the islet in the dynamical oscillator model. C) Modelled transition from activity to quiescence within the Boolean lattice resistor network model as P_exc_ is reduced, for *p* = 0.3 and *Sp* = 0.5. Note in all cases; for islets lacking gap junction coupling, with zero gap junction conductance and for *p* = 0, the transition is trivially linear (blue dashed).

In experiments with mutant K_ATP_ subunit expression, cells were considered ‘inexcitable’ if they showed GFP and Kir6.2^[ΔN30,K185Q]^ expression. In this case of endogenous heterogeneity, for a given concentration of diazoxide, we can consider a cell is ‘inexcitable’ if it is quiescent in the absence of electrical coupling. By plotting activity in the presence of coupling (representing the resultant activity) against activity in the absence of coupling (representing intrinsic cellular excitability) similar phase-transitions are apparent; with quantitative agreement between experimental data, dynamic coupled oscillator model and static network model (figure 7A–C). The phase transition in the dynamic coupled oscillator model was dependent on how heterogeneity was generated, where heterogeneity in multiple factors rather than any one factor was required for agreement with experimental data (SI figure S7). Therefore critical behavior can occur more generally from the coupling between heterogeneous cellular populations within the islet, as exemplified here experimentally and theoretically.

## Discussion

The islet of Langerhans shows unique functional properties that result from the underlying network interactions between constituent cells. One important property is that β-cells within the islet show globally quiescent behavior at lower levels of glucose despite showing a heterogeneous range of glucose sensitivities when in isolation. A proposed mechanism underlying this behavior is that at a given glucose stimulation, inactive cells suppress cells that otherwise would be active, via gap junction coupling. We applied predictive mathematical models to quantify this behavior and determined the relative role of K_ATP_ channel activity (controlling cellular excitability) and gap junction activity (controlling cellular coupling) in shaping this islet response. We then experimentally verify predicted behavior using two independent experimental models.

### Critical Behavior in Islets and Theoretical Models

In line with previous work describing coupled electrical dynamics, we showed that the structure and function of the islet cellular network can be described through principles of network theory [20], [21]. Both the Boolean network and dynamic oscillator models predict the emergent behavior upon coupling between a heterogeneous cell population. The islet rapidly transitions between globally coordinated active and inactive states upon disproportionally small changes in the excitability of the constituent cells as they approach a critical ‘threshold’ excitability. This occurs under both conditions of β-cell heterogeneity we examined: the imposed bimodal β-cell populations achieved through expression of Kir6.2^[ΔN30,K185Q]^ or Kir6.2^[AAA]^ mutations; and endogenous β-cell heterogeneity with diazoxide activation of K_ATP_.

The Boolean model reveals that there is an imbalance in the ability of excitable and inexcitable cells to respectively propagate stimulation or suppression. A low *Sp* in the model indicates a preference for excitable cells to be suppressed by inexcitable cells. This describes how gain-of-function Kir6.2^[ΔN30,K185Q]^ expressing cells (which are glucose-unresponsive) suppress activity in coupled normal cells at high glucose, and how loss-of-function Kir6.2^[AAA]^ expressing cells are suppressed by normal cells at low glucose (figure 2, 4). The role of *p* (gap junction coupling) determines the spatial extent over which suppression occurs. As shown in figure S1 and S2, a low *p* results in coupled behavior restricted to a few cells and therefore inactive cells are unlikely to couple to many active cells and mediate suppression. When *p* exceeds the critical coupling probability (∼0.25) then coupling spans the whole network and inactive cells can couple to and suppress most active cells in the network. The sharp transition that emerges upon *p*>0.25 can be understood by considering that the threshold for activity (*Sp*) is well defined with a sharp cutoff for the P_exc_ which determines whether the cluster is active or inactive. The agreement with experimental data indicates that there is little variability between cells in this threshold for suppression, as also supported by the distributions of fitted *Sp* (figure 2E).

While the coupled dynamic oscillator model also predicts and describes the phase transitions present, the Boolean model describes the essential features that govern multicellular regulation of islet excitability. Results suggest that the islet may fundamentally behave in a binary fashion in terms of gap junction coupling and K_ATP_-regulated excitability. Given the proportion of cells that intrinsically (i.e. in the absence of coupling) show activity at a given glucose stimulation and the level of coupling, the overall response of the islet can be approximated through this reductionist model. Of course dynamical features are missing from the Boolean model which is only described by the coupled dynamic oscillator model: including the altered oscillatory characteristics in the pre-critical state. The low *p* (0.30–0.38) required for the Boolean network model to quantitatively describe experimental data points to incomplete coupling present; and this can explain the residual activity in the post-critical state (figure 1C). Recent studies of coordinated [Ca^2+^]_i_ oscillations and waves in the islet have indicated a ‘backbone’ of a few strong connections dominate coupled [Ca^2+^]_i_ dynamics, which is equivalent to a similarly low *p* [19], [20]. The ability of the coupled dynamic oscillator model to also describe the transition between globally active and inactive states, suggests that the dynamics of the islet may behave according to general principles of coupled dynamical systems. Further work is needed to examine this critical behavior in more detail, including power law scaling and its dependence on network parameters and cellular properties.

The phase-transition behavior can also be explained through a mean-field theory analogy (SI figure S8). Cells expressing the mutant Kir6.2^[ΔN30,K185Q]^ are intrinsically inexcitable (figure S8A). In the ‘pre-critical’ regime the number of these cells is below a critical threshold and insufficient to suppress glucose-stimulated activity via coupling; therefore all cells are recruited to elevate [Ca^2+^]_i_. When the number of these inactive cells approaches the critical threshold (*Sp* = 0.15 for Kir6.2^[ΔN30,K185Q]^ expression) critical behavior emerges and coupling mediates suppression of other active cells. In normal islets treated with diazoxide, endogenous β-cell heterogeneity leads to variable intrinsic excitabilities and we expect diazoxide renders cells less glucose sensitive to be inexcitable (figure S8B). In the absence of coupling these are observed to be inactive (figure 6). Low concentrations of diazoxide (<50 µM) render only a few cells inexcitable, which is below the critical threshold (*Sp∼0.5*) and insufficient to suppress [Ca^2+^]_i_. At higher concentrations of diazoxide (>100 µM) more cells are rendered inexcitable, and when this number exceeds the critical threshold, coupling mediates suppression of other normally excitable cells. We predict that observed glucose-dependent activity and the coupling dependence can also be explained in this way (see below).

The *Sp* for endogenous heterogeneity is higher than that for an imposed biomodal distributions (i.e. diazoxide treatment versus Kir6.2^[ΔN30,K185Q]^ or Kir6.2^[AAA]^ expression) suggesting a more even balance between the ability of excitable and inexcitable cells to respectively propagate stimulation or suppression in wild-type islets (figure 6, 7). This balance may arise from the different distribution of heterogeneity present, but a phase-transition still emerges in the presence of coupling indicating a more general regulation of multicellular excitability.

Therefore through limited coupling of heterogeneous populations of cells, critical behavior emerges in the islet dynamical system where large changes in activity result from small changes in the constituent cellular excitabilities.

### Applicability to Physiology and Diabetes

Gap junctions impact islet behavior in two main ways. At high glucose (K_ATP_ channel-closure), gap junctions coordinate oscillatory dynamics of membrane depolarization and [Ca^2+^]_i_ to generate a robust pulsatile insulin secretion [13], [14], [32]. A number of recent studies have examined this aspect, including multicellular modelling and quantitative analyses [14], [18], [19], [21]. Equally important however is that at lower glucose (K_ATP_ channel-opening), gap junctions mediate a suppression of membrane depolarization, [Ca^2+^]_i_, and insulin secretion [10], [13], [17]. The mechanisms involved in mediating suppression are not well characterized, and several experimental perturbations have yielded unexpected results or have not been well described theoretically [17], [34], [35]. Here, we were able to quantitatively describe suppressive behavior resulting from coupling, which yields a more complete understanding for how the islet functions under conditions of K_ATP_ channel opening.

At 6–7 mM glucose, the islet sharply transition between global quiescence and globally synchronized [Ca^2+^]_i_ oscillations. In the absence of coupling, the progressive elevation in the number of cells showing [Ca^2+^]_i_ elevations is gradual [10]. This follows the same behavior as variable Kir6.2^[ΔN30,K185Q]^ expression and diazoxide concentration (figures 2,6). At <6 mM glucose, global suppression is equivalent to >15% Kir6.2^[ΔN30,K185Q]^ expression or >100 µM diazoxide; whereas at >7 mM glucose global activity is equivalent to <15% Kir6.2^[ΔN30,K185Q]^ expression or <50 µM diazoxide. The 6–7 mM glucose transition is therefore equivalent to behavior at ∼15% Kir6.2^[ΔN30,K185Q]^ expression or 50–100 µM diazoxide. As such, we propose results from the Boolean model, as illustrated by the mean-field theory analogy, have greater implications by describing physiological glucose-dependent islet electrical activity (figure S8C). Coupling heterogeneity and islet architecture lead to variability in the number and strength of connections, impacting the phase transition. These factors may therefore play a role in shaping the physiological regulation of glucose-stimulated [Ca^2+^]_i_ and insulin secretion (Figures S5,S6).

At 11 mM glucose, heterogeneity leads to a small population of β-cells (<10%) remaining inactive in the absence of coupling [10]. In the presence of coupling there is global activity with a lower plateau fraction compared to higher glucose levels (e.g. 20 mM) [36]. This matches the behavior at 5–10% Kir6.2^[ΔN30,K185Q]^ expression or 50 µM diazoxide in the respective absence and presence of coupling. Therefore an alternative view for how oscillatory dynamics are shaped at an islet-wide level is that less-active cells within the β-cell network have a modulatory effect on overall oscillation waveform, rather than oscillations being shaped by purely intrinsic properties of the β-cells. Importantly, the reduced plateau fraction of [Ca^2+^]_i_ bursts at 5–10% Kir6.2^[ΔN30,K185Q]^ expression correlates with a significant decrease in insulin secretion (figure 3). A decrease in burst duration has previously been suggested to reduce insulin release [37], as supported by these results. Thus subtle alterations in the balance of constituent cell excitabilities have a strong physiological effect on islet function.

Our results also have implications for neonatal diabetes mellitus (NDM), where the majority of cases result from mutations to Kir6.2 or SUR1 K_ATP_ channel subunits [8], [38]. Kir6.2^[ΔN30,K185Q]^ expression models this disease [27]. Our results show that NDM mutations gives rise to a disproportionate suppression in [Ca^2+^]_i_ and insulin release, thereby causing diabetes due to the critical behavior that emerges from coupling and network dynamics. This also explains how an absence of coupling elevates [Ca^2+^]_i_ and insulin release (figures 1, 6) to prevent the progression of diabetes. This was experimentally demonstrated in a recent study [26], and the rescue of diabetes can only be understood mechanistically at the multicellular level. Clearly in human diabetes, mutations are not expressed mosaically. However, the diazoxide results which depend on a continuum of heterogeneity (figure 6) demonstrate that critical behavior exacerbates NDM upon uniform K_ATP_ channels overactivity. Other monogenic diabetes causing mutations that affect β-cell excitability, such as Glucokinase [6], may also have similar effects on islet excitability and lend themselves to analysis by the Boolean model and coupled oscillator model.

Mutations causing NDM are functionally equivalent to >15% Kir6.2^[ΔN30,K185Q]^ or >100 µM diazoxide, effectively residing in a post-critical state suppressing global [Ca^2+^]_i_. There exists a spectrum of K_ATP_ channel mutations linked to diabetes, where weaker mutations to Kir6.2 and SUR1 elevate the risk of type2 diabetes [39]–[41]. These mutations likely have a more subtle effect on islet excitability and as a consequence we predict that islets residing in the pre-critical state (<15% Kir6.2^[ΔN30,K185Q]^ or <50 µM diazoxide) would still be susceptible to diabetes following metabolic stress. Further, while gap junction reduction recovers insulin release and glucose control in the post-critical regime (i.e. NDM), we predict a gap junction increase would be beneficial in the pre-threshold regime (i.e. type2 diabetes).

Converse to this, results from Kir6.2^[AAA]^ islets show how critical behavior provides the islet with a resilience to over-excitable β-cells. Given the ∼85% threshold of excitable cells required to elevate [Ca^2+^]_i_, with ∼70% over-excitable Kir6.2^[AAA]^-expressing cells, many of the ∼30% normal β-cells would also need to be active (e.g. >50% at ∼5.5 mM glucose [10]). This explains only the minor shift in glucose-stimulated [Ca^2+^]_i_ that occurs following K_ATP_ inactivity and highlights the role electrical coupling plays in protecting islets against hyper-excitability [17].

Therefore we describe the emergence of critical behavior linking multiple levels including molecular and cellular behavior, multicellular behavior, *in-vivo* physiology, disease and treatment.

### Implications for General Dynamical Systems

This study also has implications for general understanding of physiological systems composed of coupled dynamic units. Previous theoretical studies have shown how the introduction of non-oscillatory elements above a critical level in a generalized coupled oscillator system can lead to cessation of global oscillations with phase transitions [42], [43]. Our study experimentally and theoretically demonstrates this in a disease relevant system. Further, prior studies theoretically demonstrated that the fraction of excitable elements (i.e. P_exc_) and coupling strength (i.e. *p*) exist in a phase plane where increased coupling decreases the number of inactive elements required for suppression [43], [44]. We demonstrated this experimentally and theoretically, with Kir6.2^[ΔN30,K185Q]^ and diazoxide-induced suppression. While strong coupling promotes robust synchronization, it will increase suppression from non-oscillatory inexcitable units. Given a small population of inactive β-cells exists as a result of cellular heterogeneity, these generalized theoretical studies imply inappropriate elevations in coupling would be deleterious, by reducing glucose-stimulated [Ca^2+^]_i_. As such the level of coupling is likely at an optimal level to balance global synchronization and suppression.

The strong link between dynamical β-cell networks and generalized coupled oscillators implies similar behavior can be expected in other physiological systems. In the heart, electrical activity is initiated by pacemaker cells and propagates to excite contractile myocytes. In culture, non-excitable fibroblasts proportionally reduced cardiomyocyte wave propagation bursts frequency with Cx43 dependence [45]. No activity was reported for >30% fibroblast penetrance and modulation of action potential frequency occurred at <30% fibroblast penetrance (implying *Sp* = 0.3). While the mechanisms of coupling-dependent suppression are very different compared to our study, global responses are similar implying similar governing principles. Similarly, pacemaker cells exhibit dominance over myocytes at an optimal gap junction conductance [46]; where high coupling leads to arrhythmias and low coupling leads to poor synchronization [47]. Neurons also display intrinsic oscillatory behavior, and the effects of coupling and presence of inhibitory and excitatory neurons on synchronization and phase modulation is an active area of research [48]–[53]. Critical dynamics have been described theoretically to emerge from excitatory and inhibitory units in neuronal networks [54], and a computational study which introduced ‘contrarian elements’ into neural coupled oscillator networks found that a similar threshold of 15% suppressed global dynamics [49].

We also anticipate that critical behavior resulting from coupling of heterogeneous units may be considered a general regulatory mechanism. Many systems respond to a stimulus by transitioning between inactive and active states (e.g. contractile, hormone-secretory). Our study implies that constituent cellular units need not themselves have a uniform or robust response to generate a robust multicellular response. Rather, a robust response can emerge from coupling a heterogeneous collection of cells; where coupling and architecture need only have sufficient strength and connection number *on average*. This makes the overall system robust against noise and variability, and loosens the requirement for tight regulatory mechanisms within the constituent cells. Similarly, given a constant stimulus, a robust transition between globally active and inactive states could be achieved by remodeling connectivity with a small number of inhibitory units. For example, down-regulating connections from ∼20% to ∼5% inhibitory cells would transition the system from inactive to active requiring minimal system remodeling. This also suggests how inappropriate changes in coupling or constituent cells may lead to global non-responsiveness and disease. We speculate these principles may apply to other neuroendocrine cell systems such as GH-cells or the adrenal medulla, where functional remodeling elevates hormone secretion upon physiological stimuli [18], [55]. Indeed many of these principles have been linked with GnRH neuron function during development [56], [57]. Therefore we suggest that new and robust functionalities can be generated at the multicellular level from the coupling of non-robust constituent cell function, requiring minimal system resources compared to the requirements were cells to act autonomously.

### Conclusions

Any living system cannot avoid deterioration through mutation or other pathological insult. This study experimentally and theoretically demonstrates that if the fraction of inactive elements exceeds a coupling-dependent threshold, the global activity of the system can be abolished. In the case of the islet this explains how inactive cells can suppress the activity of other cells, thereby preventing the secretion of insulin. In the case of K_ATP_ mutations, this quantifies the threshold of inexcitable cells required for pathogenic symptoms and explains how coupling can eliminate the emergence of diabetes or exacerbate it. Overall, this gives a new understanding for how emergent properties of the islet as a β-cell network are generated; as well as for understanding islet dysfunction in diabetes and novel ways to overcome dysfunction. More broadly, this generates insight into emergent behavior of multicellular systems in general.

## Methods

### Ethics Statement

All experiments were performed in compliance with the relevant laws and institutional guidelines, and were approved by the University of Colorado Institutional Biosafety Committee (IBC) and Institutional Animal Care and Use Committee (IACUC).

### Animal Care

The generation of Rosa26-Kir6.2^[ΔN30,K185Q]^ (‘gain-of-function’ K_ATP_ subunit with GFP co-expression), Pdx-Cre^ER^ (β-cell specific inducible Cre), Kir6.2^[AAA]^ (‘loss-of-function’ K_ATP_ subunit with GFP tag), and Cx36^−/−^ (Connexin36 global knockout) have been described previously [27], [28], [58], [59]. Expression of variable Kir6.2^[ΔN30,K185Q]^ was achieved in β-cells by crossing Rosa26-Kir6.2^[ΔN30,K185Q]^ and Pdx-Cre^ER^ mice, and inducing Kir6.2^[ΔN30,K185Q]^ expression in 8–16 week old mice by 1–5 daily doses of tamoxifen (50 mg/g body-weight). Littermates lacking Rosa26-Kir6.2^[ΔN30,K185Q]^ and/or Pdx-Cre^ER^ were used as controls.

### In-Vivo Measurements

Blood glucose was measured daily and averaged over day 27–29 post tamoxifen induction using a glucometer (Ascensia Contour, Bayer). Plasma insulin was measured at day 29 from blood samples centrifuged for 15 minutes at 13,900RCF, and assayed using mouse ultrasensitive insulin ELISA (Alpco).

### Islet Isolation and Insulin Secretion

Islets were isolated by collagenase injection into the pancreas through the pancreatic duct; the pancreas was harvested and digested, and islets were handpicked [23]. Islets were maintained in RPMI medium (Invitrogen) supplemented with 10% FBS, 11 mM glucose, 100 U/ml penicillin, 100 µg/ml streptomycin, at 37°C under humidified 5% CO_2_ for 24–48 hours prior to study. For insulin secretion measurements, islets (5/column, duplicates) were pre-incubated in Krebs-Ringer buffer (128.8 mM NaCl, 5 mM NaHCO_3_, 5.8 mM KCl, 1.2 mM KH_2_PO_4_, 2.5mM CaCl_2_, 1.2 mM MgSO_4_, 10 mM HEPES, 0.1% BSA, pH 7.4) plus 2 mM glucose; then incubated for 60 minutes in Krebs-Ringer buffer plus 20 mM glucose. After incubation, the medium was sampled and insulin concentration assayed using mouse ultrasensitive insulin ELISA. To estimate insulin content, islets were lysed in 1% TritonX-100 and frozen at −20°C overnight.

### Calcium Imaging

To measure [Ca^2+^]_i_ dynamics, isolated islets were loaded with 4 µM FuraRed-AM (Invitrogen) in imaging medium (125 mM NaCl, 5.7 mM KCl, 2.5 mM CaCl_2_, 1.2 mM MgCl_2_, 10 mM Hepes, 2 mM glucose, and 0.1% BSA, pH 7.4) at room temperature for 90–120 minutes and held in polymdimethylsiloxane (PDMS) microfluidic devices [17]. FuraRed fluorescence was imaged on a spinning-disk confocal microscope (Marianas, 3I) with a 40× 1.3NA Plan-NEOFluar oil-immersion objective (Zeiss) maintained at 37°C. Images were acquired at 1 frame/sec using a 488 nm diode laser for excitation and a 580–655 nm long-pass filter for emission. Time-courses were acquired 10 minutes after change in glucose concentration, diazoixide or 18-α-glycyrrhetinic acid application. Time-courses are displayed as normalized to the average fluorescence.

### Data Analysis

All images were analyzed using custom MATLAB (Mathworks) routines or using Slidebook (3I). To calculate islet activity, images were smoothed using a 5×5 average filter. The variance of pixel time-courses was first calculated for a quiescent reference cell; manually selected from an area which displayed no fluctuations in intensity over time compared with image noise. A pixel was considered ‘active’ if its time-course showed a variance >2 standard deviations above the variance of the quiescent reference cell [10], [20]. Photobleaching was accounted for through a linear fit, and time-courses were rejected if excessive motion artifacts occurred. The area of active cells in terms of pixels, was determined for each condition and expressed as a fraction of total islet pixel area as defined by mean FuraRed fluorescence. GFP^+^ regions were defined as having a mean pixel fluorescence intensity above that measured in GFP^−^ wild-type cells. The area of active cells in GFP^+^ regions was expressed as a fraction of the total GFP^+^ area. Information describing activity is represented as a false-color HSV image where Hue is set to 1 (red), Saturation is set to 1 for active cells and 0 (no color) for inactive cells, and Value (intensity) is set to the average FuraRed fluorescence. Data are presented as mean±SEM. For comparison of two means, Student's t-test was utilized. For comparison of multiple means, ANOVA was utilized along with Tukey's HSD test.

### Boolean Network (Percolation) Model of Suppression

Bond percolation is a sub-model of percolation theory [31], [60] which can be used to simulate the islet [14], [20], [21]. For a lattice of nodes (cells) in a given geometry, adjacent nodes are connected with a ‘coupling probability’ *p*, or not connected with a probability (1-*p*). Connected nodes are considered ‘functionally coupled’, where activity is synchronized at high glucose and suppression mediated at low glucose. We implemented simulations of bond percolation lattices (figure S1) as previously described [20]. Briefly, cubic lattices with alternating node and bond sites (length L = 11) were generated. Probabilities were assigned to each bond site with a uniform distribution (0 to 1). Neighboring nodes were coupled if the bond site probability was less than or equal to the coupling probability *p*. Clusters of coupled nodes were identified and potential bond sites removed to establish a matrix of identified coupled nodes (figure S1A).

Coupling-mediated suppression is based on the principle that a threshold fraction of non-responsive (‘inexcitable’) cells can suppress all other cells to which they are coupled [17], [20]. Within a cluster of coupled nodes, if the fraction of inexcitable cells is greater than a threshold fraction (*Sp*), then all cells within the cluster are inactive. Experimental studies indicate this threshold is <30% [17] and has been modelled to be ∼15% for MIN6 aggregates [20]. The probability P_exc_ defines the fraction of cells within the islet that are intrinsically excitable; where in the absence of coupling they would be active. Within each cluster of coupled nodes a binomial distribution was used to estimate the probability of there being a threshold number of inactive cells to lead to suppression. Given a threshold number of inactive cells required for suppression (*k*), a total number of cells in a cluster (*n*), and the fraction of inactive cells (*q*); the probability that a coupled cluster is active (Pr) is:
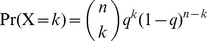
(1)where
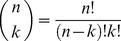
(2)


The cumulative distribution for *k* to *n*, P(X≤*k*), represents the probability of suppression (sufficient inexcitable cells) in a coupled cluster. To obtain the resultant % activity, P(X≤*k*) was averaged over all clusters within the islet, weighted by the number of cells *n* in each cluster. ‘*k*’ normalized to ‘*n*’ gives the fraction of inexcitable cells required for suppression (*Sp*). ‘1-*q*’ gives the fraction of excitable cells (P_exc_). 500 simulations were run for each P_exc_ = [0 1] and *p* = [0 1], at 0.01 increments for given values of *Sp*. The *p* and *Sp* parameters that generated the best fit for the simulation mean to experimental data were determined by a chi-squared minimization. To determine the probability distribution for *p* and *Sp*, 4000 simulations were run and each simulation was separately fitted for *p* and *Sp* by chi-squared minimization.

### Coupled Dynamical Model

The islet model was based on the Cha-Noma β-cell model [29], [30], itself based on the Fridlyand β-cell model [61], [62], and adapted to include cell-cell coupling. We also included further aspects of cell-cell coupling and altered K_ATP_ channel function. A list of parameters used in the model is included in SI Table S1. The membrane potential (*V_i_*) of each β-cell *i* is related to the total transmembrane current as:
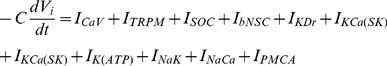
(3)


Where the kinetics of each current is described in [29], [30].

To simulate gap junction coupling and a multicellular islet, multiple ‘cells’ were simulated with a coupling current between each neighboring cell. The membrane potential for each cell *i* was modified to account for coupling to *j* neighboring cells:
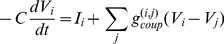
(4)


Heterogeneity in coupling was introduced by randomly assigning the gap junction conductance 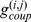 between cells *i* and *j*, according to an experimentally measured distribution [unpublished data], with SD/mean = 70%. To more accurately model β-cell coupling architecture, random cell lattices were created using a position- and availability-based sphere-packing algorithm (mean,SD number of cell-cell connections = 5.3,1.7) [63] (figure S3).

*I_K(ATP_*_)_ was described in [29], [30] as:
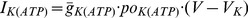
(5)


Where 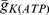 is the open channel conductance and 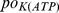 represents the mean open probability which is given by:
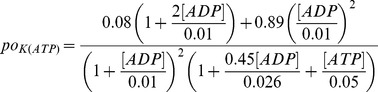
(6)


Endogenous heterogeneity was modelled by randomizing all parameters indicated in Table S1 between cells about a mean value according to a Gaussian distribution with SD/mean = 10%. To generate heterogeneity in electrical responses equivalent to experimental measurements, the open channel conductance 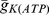 was randomized between cells about a mean value according to a Gaussian distribution with SD/mean = 25%. This heterogeneity achieves variability in activity that matches experimental measurements in islets lacking Cx36 [10], [14].

To model Kir6.2^[ΔN30,K185Q]^ expression, the open probability 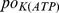 was modified in a proportion (P_exc_) of simulated cells:
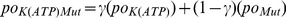
(7)where *γ* is a constant representing the fraction of ATP-insensitive current (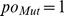), and was set to 0.5.

To model diazoxide treatment, the fraction of ATP-insensitive current was increased in all cells uniformly according to:
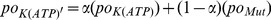
(8)such that α = 1 represents an untreated islet, and α = 0.5 is equivalent to 100% expression of Kir6.2^[ΔN30,K185Q]^.

All simulations were initially written and verified in MATLAB, then rewritten in C++ and simulated on the University of Colorado JANUS supercomputer. The model was solved using a constant time-step Euler integration scheme (Boost C++ Libraries) with 100 µs step-time and 100 ms sampling-time. Rendering simulations was performed with Mathematica 9.0 (Wolfram Research).

## Supporting Information

Figure S1 Description for how the Boolean network model is constructed and how the network parameters *p*, P_exc_ affects network activity. A) Generation of coupled network and identification of connected clusters within the network. A 3 dimensional array of nodes (cells) are generated (in this case an L = 11 lattice). Bond sites between neighboring nodes are populated according to the coupling probability *p*, and nodes that belong in connected clusters identified. The number of nodes in each connected cluster of the network is then recorded. B) Histogram for the density of nodes as a function of identified cluster sizes for different values of *p*. Below ∼0.25 only very small clusters are present. Above ∼0.25 a single large connected cluster emerges to which the majority of cells belong. Data are averaged over 5000 simulations. C) Dependence of cluster activity on the cluster size for two different values of P_exc_, where *Sp* = 0.15. When P_exc_<(1-Sp) increasing cluster size leads to reduced activity, whereas when P_exc_>(1-Sp) increasing cluster size leads to increased activity. This explains the respective low and high activity in figure 1B. Data are averaged over 5000 simulations. Overall, the coupling probability *p* (representing gap junction strength) determines the distribution of connected cluster sizes within the islet network. The P_exc_ value (representing cellular excitability) then determines how active each connected cluster is depending on its size. The combination of these 2 factors then determines the overall network activity.
(TIF)

Figure S2 The effect of functional coupling parameter *p* on the activity of simulated Boolean networks and comparison with percolation theory, see [Hraha, T.H., et al., *Biophys J*, 2014. **106**(1): p. 299–309]. A.) Percent activity of L = 11 lattices was simulated for P_exc_ = 70% for *p* = 0.1 (*p*<*p_c_* = 0.2488 for cubic lattice, i.e. sub-critical coupling) and *p* = 0.3 (*p*>p_c_, i.e. supra-critical coupling). B.) Three-dimensional false color maps of network activity in representative L = 31 lattices for *p* = 0.1 and *p* = 0.3, for P_exc_ indicated in A. C) Mean size of largest connected clusters as a fraction of 3D network in simulated networks as a function of *p*. D.) Three-dimensional false color maps showing distinct ‘clusters’ of coupled cells in simulated Boolean networks with *p* = 0.2 (*p*<*p_c_*) and *p* = 0.3 (*p*>*p_c_*), as indicated in C. Note for *p* = 0.1, the majority of clusters approach single nodes (cells).
(TIF)

Figure S3 Penetrance of Kir6.2^[ΔN30,K185Q]^ and Kir6.2^[AAA]^. Representative GFP images showing the presence of mutant Kir6.2 expressing cells as indicated by GFP co-expression (for Kir6.2^[ΔN30,K185Q]^) or a GFP tag (for Kir6.2^[AAA]^). GFP tagged cells are constitutively inactive (inexcitable) in the case of Kir6.2^[ΔN30,K185Q]^ and constitutively active for Kir6.2^[AAA]^. As such P_exc_ which represents the number of excitable cells is equal to 100%-%GFP upon Kir6.2^[ΔN30,K185Q]^ expression and is equal to %GFP for Kir6.2^[AAA]^ expression. For Kir6.2^[ΔN30,K185Q]^ expressing islets, controlled expression is induced through variable doses of tamoxifen injections, for which representative images are shown for wild-type (GFP = 0%, P_exc_ = 100%), pre-critical (GFP = 10%, P_exc_ = 90%), critical (GFP = 20%, P_exc_ = 80%), and post-critical (GFP = 55%, P_exc_ = 45%), conditions of islet activity. Conversely, Kir6.2^[AAA]^ expressing islets show on average ∼70% penetrance, such that P_exc_ = 70%.
(TIF)

Figure S4 [Ca^2+^]_i_ in Kir6.2^[ΔN30,K185Q]^ expressing and non-expressing cells within the islet as indicated by GFP coexpression. A) Percent cells showing [Ca^2+^]_i_ elevations in GFP positive or GFP negative cells as a function of P_exc_ (100-%GFP). Green diamonds indicate those cells expressing GFP whereas black squares indicate those cells lacking GFP. B) Mean(±s.e.m.) percent cells showing [Ca^2+^]_i_ elevations in GFP positive or GFP negative cells for data binned to wild-type, pre- and post-critical ranges, as determined by %GFP. *indicates significant difference (p<0.05) between data as indicated.
(TIF)

Figure S5 Effects of islet architecture on phase transition behavior. A) Schematic representations of the cubic lattice, and B) representative example of the quasi-spherical sphere packing architectures used for simulating the dynamical oscillator model. C) Comparison of simulated islet activity as a function of percent excitable cells (P_exc_) for cubic and sphere packing architectures in the dynamical model for physiological wild-type gap junction conductance.
(TIF)

Figure S6 Effects of endogenous cellular heterogeneity and coupling heterogeneity on phase transition behavior. A) Simulated islet activity as a function of percent excitable cells (P_exc_) in the presence and absence of heterogeneous distributions of gap junction coupling conductance in the dynamical model. B.) Simulated islet activity as a function of percent excitable cells (P_exc_) in the presence and absence of heterogeneous distributions of cell physiology parameters in the dynamical model.
(TIF)

Figure S7 Dependence of phase transitions in β-cell network activity on the origin of endogenous β-cell heterogeneity in simulated islets. A) Left: Percent cells showing [Ca^2+^]_i_ elevations in simulated islet as a function of a uniform increase in the fraction of ATP-insensitivity of K_ATP_ channel activation (α) across cells of the islet. Heterogeneity is present in all parameters described in Table S1 and as used elsewhere in this study. Mean simulation data is presented for zero gap junction conductance (0 pS) and wild-type gap junction conductance (120 pS). Right: activity of fully-coupled islet system as a function of activity in the uncoupled islet systems which represents the excitability of the constituent cells, with heterogeneity present in all parameters. For islets lacking gap junction coupling, with zero gap junction conductance, the result is trivially linear (dashed). B) As in A for heterogeneity solely in K_ATP_ channel activity. C) As in A for heterogeneity solely in glycolytic flux.
(TIF)

Figure S8 Mean-field theory analogy of β-cell network activity. The excitability of constituent units (i.e. their glucose sensitivities) and the resulting network activity takes into account coupling and different experimental perturbations. A) Cells of wild-type islets are inexcitable at low glucose (2 mM) and all excitable at high glucose (20 mM), therefore wild-type islets are respectively fully inactive and fully active. Cells expressing Kir6.2^[ΔN30,K185Q]^ are glucose-insensitive and constitutively inexcitable. When Kir6.2^[ΔN30,K185Q]^ penetrance is <15% (P_exc_>0.85) there are insufficient inexcitable cells to suppress global activity, and so coupling leads to inexcitable cells being recruited to be active. However, when Kir6.2^[ΔN30,K185Q]^ penetrance rises above ∼15% (P_exc_<0.85), global quiescence ensues where the majority of normally excitable cells are rendered inactive. B) For low diazoxide (<50 µM), there are fewer excitable cells compared to untreated, however if coupling exists then all cells are recruited to be active. However, in the absence of coupling the resulting activity is the same as the composition, which results in lower activity. In the case of higher diazoxide treatments (>100 µM) the proportion of inexcitable cells exceeds the threshold for suppression. If coupling exists then all cells are rendered inactive. However in the absence of coupling the resulting activity is the same as the composition and some cells remain active. C) This mechanism can also explain how the islet maintains a robust well-defined glucose-stimulated response, but not in the absence of gap junction coupling. As glucose is increased more cells become excitable, but in the presence of coupling if the excitable fraction is less than a critical threshold (e.g. at ∼5 mM glucose), all cells are rendered inactive and the islet is quiescent. At a glucose level where the excitable fraction exceeds the critical threshold (e.g. ∼11 mM glucose) all cells are recruited to be active. However in the absence of coupling the activity again reflects the excitability composition within the islet: under conditions where normal coupling leads to quiescence greater activity occurs, but under conditions where normal coupling leads to global activity reduced activity occurs.
(TIF)

Movie S1 Representative movie of experimentally measured [Ca^2+^]_i_ for control conditions. Data is equivalent to that in figure 2B, panel I. Note decreases in FuraRed fluorescence are equivalent to [Ca^2+^]_i_ increases. Movie played at ×20 speed.
(AVI)

Movie S2 Representative movie of experimentally measured [Ca^2+^]_i_ for ‘pre-critical’ conditions. Data is equivalent to that in figure 2B, panel II. Note decreases in FuraRed fluorescence are equivalent to [Ca^2+^]_i_ increases. Movie played at ×20 speed.
(AVI)

Movie S3 Representative movie of experimentally measured [Ca^2+^]_i_ for ‘critical’ conditions. Data is equivalent to that in figure 2B, panel III. Note decreases in FuraRed fluorescence are equivalent to [Ca^2+^]_i_ increases. Movie played at ×20 speed.
(AVI)

Movie S4 Representative movie of experimentally measured [Ca^2+^]_i_ for ‘post-critical’ conditions. Data is equivalent to that in figure 2B, panel IV. Note decreases in FuraRed fluorescence are equivalent to [Ca^2+^]_i_ increases. Movie played at ×20 speed.
(AVI)

Movie S5 Representative movie of simulated [Ca^2+^]_i_ for control conditions. Data is equivalent to that in figure 5B, panel I. Movie played at ×10 speed.
(AVI)

Movie S6 Representative movie of simulated [Ca^2+^]_i_ for ‘pre-critical’ conditions. Data is equivalent to that in figure 5B, panel II. Movie played at ×10 speed.
(AVI)

Movie S7 Representative movie of simulated [Ca^2+^]_i_ for ‘critical’ conditions. Data is equivalent to that in figure 5B, panel III. Movie played at ×10 speed.
(AVI)

Movie S8 Representative movie of simulated [Ca^2+^]_i_ for ‘post-critical’ conditions. Data is equivalent to that in figure 5B, panel IV. Movie played at ×10 speed.
(AVI)

Table S1 Table of new and revised parameters for the dynamic oscillator model, where all nomenclature is consistent with the previously published single cell β-cell model, see [29]. *Heterogeneity is based on Gaussian variability about the mean value with standard deviation set as percentage of the given value.
(PDF)
