# Supplementary figures and images for: Phase Transitions in the Multi-cellular Regulatory Behavior of Pancreatic Islet Excitability
Source: PLoS Comput Biol. 2014 Sep 4;10(9):e1003819. doi: 10.1371/journal.pcbi.1003819 (PMC4154652; doi:10.1371/journal.pcbi.1003819)

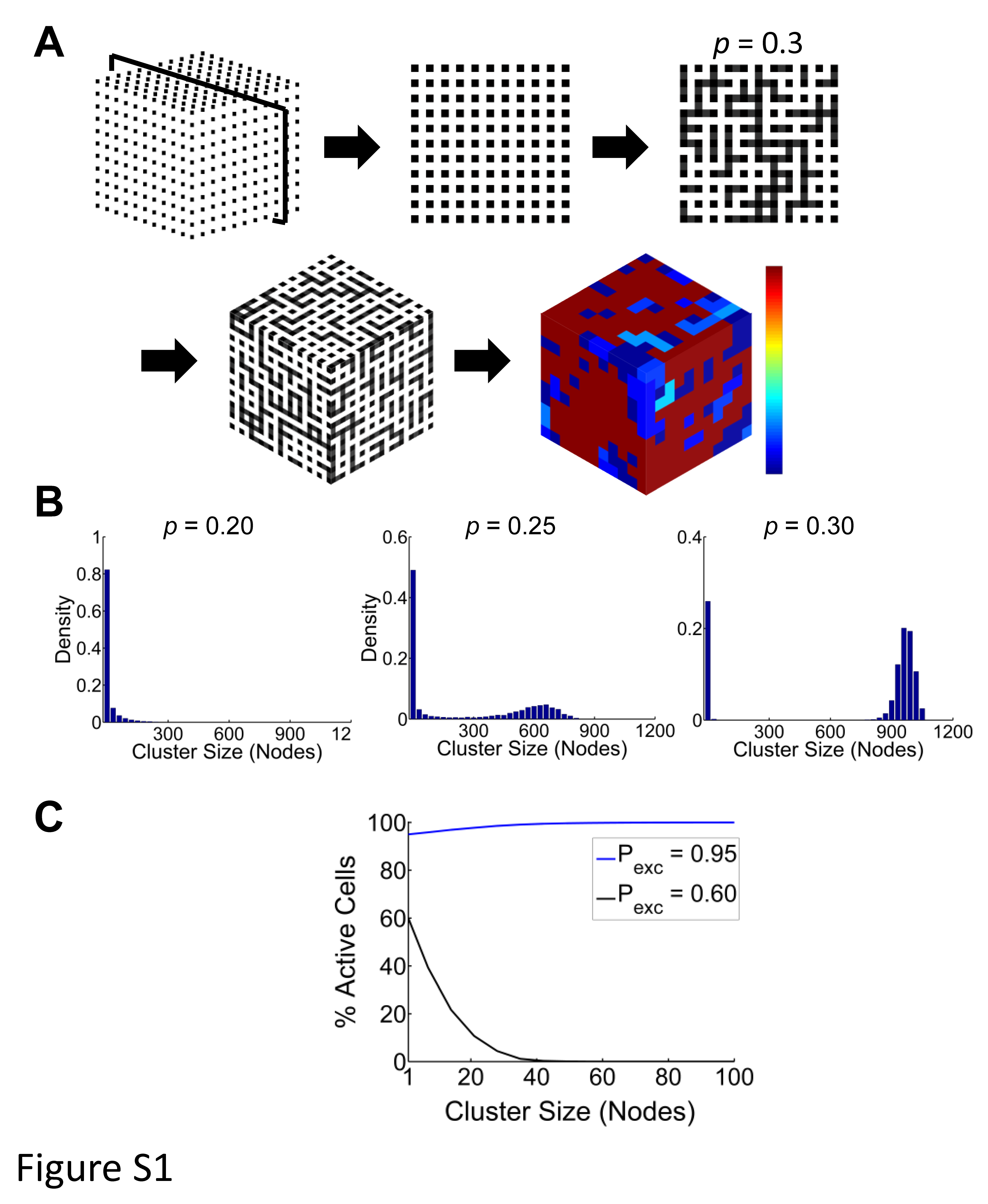

Supplement: Figure S1 — Description for how the Boolean network model is constructed and how the network parameters p, Pexc affects network activity. A) Generation of coupled network and identification of connected clusters within the network. A 3 dimensional array of nodes (cells) are generated (in this case an L = 11 lattice). Bond sites between neighboring nodes are populated according to the coupling probability p, and nodes that belong in connected clusters identified. The number of nodes in each connected cluster of the network is then recorded. B) Histogram for the density of nodes as a function of identified cluster sizes for different values of p. Below ∼0.25 only very small clusters are present. Above ∼0.25 a single large connected cluster emerges to which the majority of cells belong. Data are averaged over 5000 simulations. C) Dependence of cluster activity on the cluster size for two different values of Pexc, where Sp = 0.15. When Pexc<(1-Sp) increasing cluster size leads to reduced activity, whereas when Pexc>(1-Sp) increasing cluster size leads to increased activity. This explains the respective low and high activity in figure 1B. Data are averaged over 5000 simulations. Overall, the coupling probability p (representing gap junction strength) determines the distribution of connected cluster sizes within the islet network. The Pexc value (representing cellular excitability) then determines how active each connected cluster is depending on its size. The combination of these 2 factors then determines the overall network activity. (TIF) [file pcbi.1003819.s001.tif]

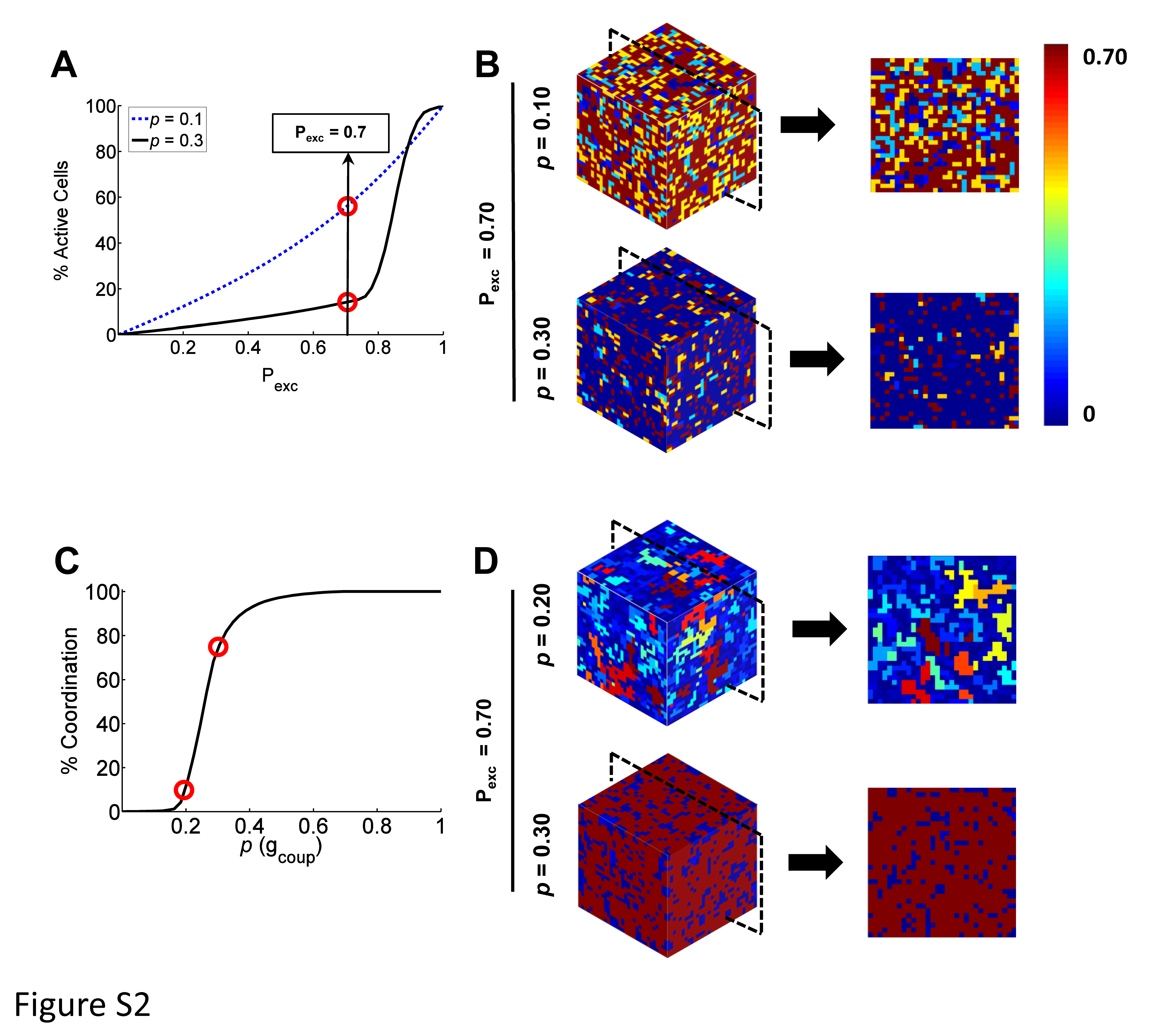

Supplement: Figure S2 — The effect of functional coupling parameter p on the activity of simulated Boolean networks and comparison with percolation theory, see [Hraha, T.H., et al., Biophys J, 2014. 106(1): p. 299–309]. A.) Percent activity of L = 11 lattices was simulated for Pexc = 70% for p = 0.1 (ppc, i.e. supra-critical coupling). B.) Three-dimensional false color maps of network activity in representative L = 31 lattices for p = 0.1 and p = 0.3, for Pexc indicated in A. C) Mean size of largest connected clusters as a fraction of 3D network in simulated networks as a function of p. D.) Three-dimensional false color maps showing distinct ‘clusters’ of coupled cells in simulated Boolean networks with p = 0.2 (p<pc) and p = 0.3 (p>pc), as indicated in C. Note for p = 0.1, the majority of clusters approach single nodes (cells). (TIF) [file pcbi.1003819.s002.tif]

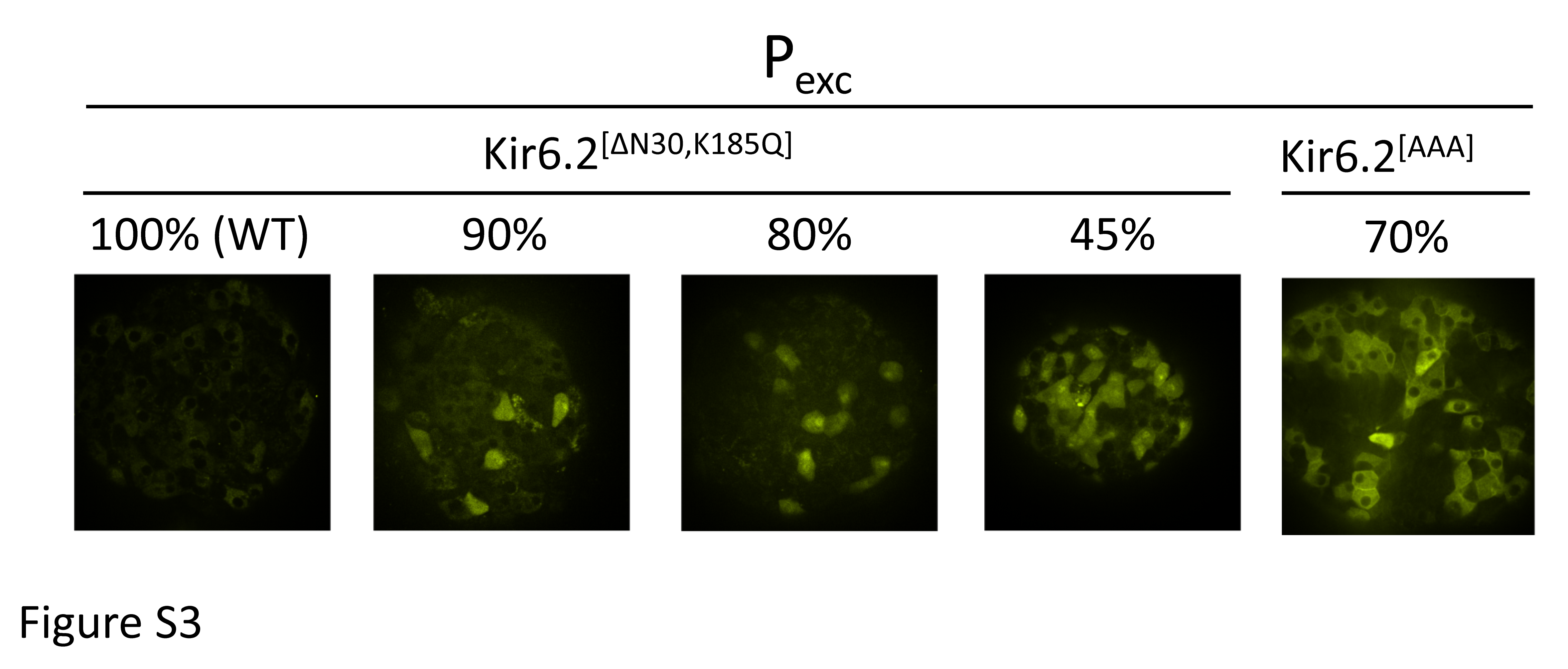

Supplement: Figure S3 — Penetrance of Kir6.2[ΔN30,K185Q] and Kir6.2[AAA]. Representative GFP images showing the presence of mutant Kir6.2 expressing cells as indicated by GFP co-expression (for Kir6.2[ΔN30,K185Q]) or a GFP tag (for Kir6.2[AAA]). GFP tagged cells are constitutively inactive (inexcitable) in the case of Kir6.2[ΔN30,K185Q] and constitutively active for Kir6.2[AAA]. As such Pexc which represents the number of excitable cells is equal to 100%-%GFP upon Kir6.2[ΔN30,K185Q] expression and is equal to %GFP for Kir6.2[AAA] expression. For Kir6.2[ΔN30,K185Q] expressing islets, controlled expression is induced through variable doses of tamoxifen injections, for which representative images are shown for wild-type (GFP = 0%, Pexc = 100%), pre-critical (GFP = 10%, Pexc = 90%), critical (GFP = 20%, Pexc = 80%), and post-critical (GFP = 55%, Pexc = 45%), conditions of islet activity. Conversely, Kir6.2[AAA] expressing islets show on average ∼70% penetrance, such that Pexc = 70%. (TIF) [file pcbi.1003819.s003.tif]

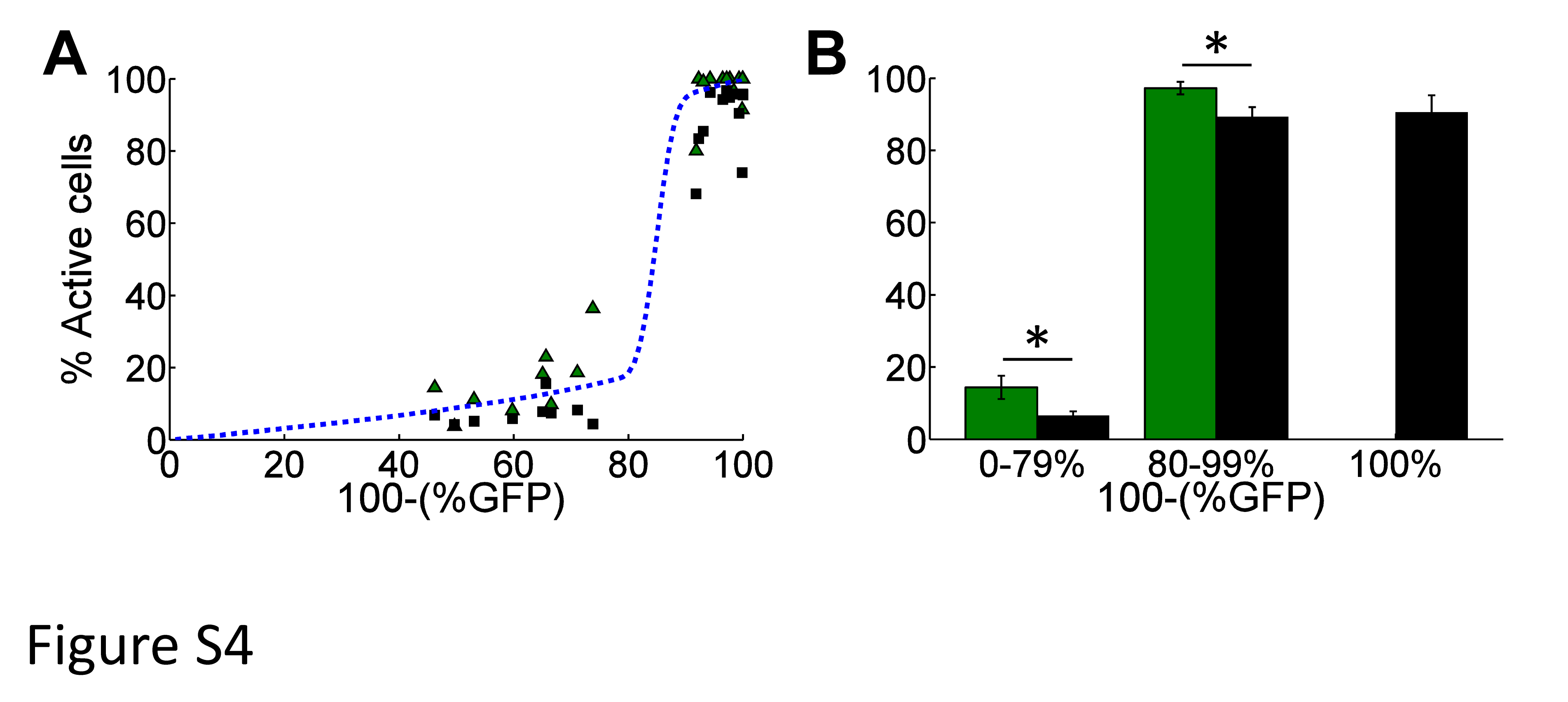

Supplement: Figure S4 — [Ca2+]i in Kir6.2[ΔN30,K185Q] expressing and non-expressing cells within the islet as indicated by GFP coexpression. A) Percent cells showing [Ca2+]i elevations in GFP positive or GFP negative cells as a function of Pexc (100-%GFP). Green diamonds indicate those cells expressing GFP whereas black squares indicate those cells lacking GFP. B) Mean(±s.e.m.) percent cells showing [Ca2+]i elevations in GFP positive or GFP negative cells for data binned to wild-type, pre- and post-critical ranges, as determined by %GFP. *indicates significant difference (p<0.05) between data as indicated. (TIF) [file pcbi.1003819.s004.tif]

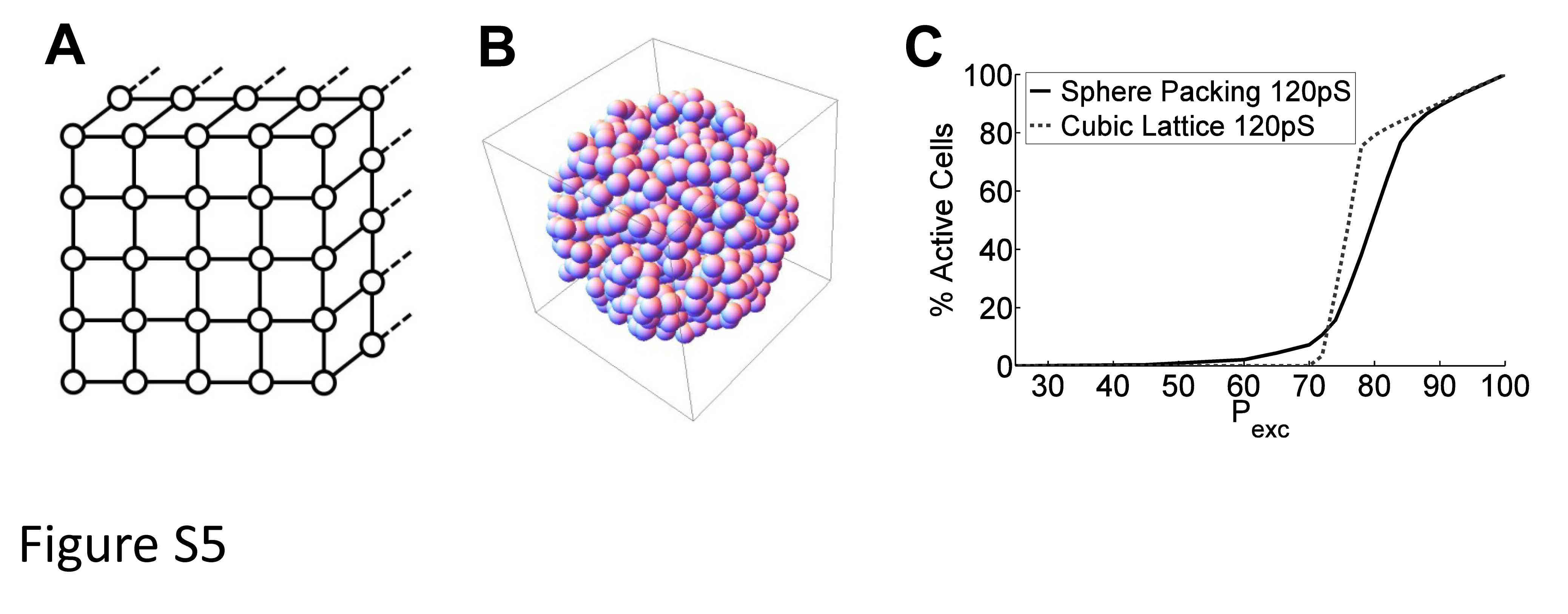

Supplement: Figure S5 — Effects of islet architecture on phase transition behavior. A) Schematic representations of the cubic lattice, and B) representative example of the quasi-spherical sphere packing architectures used for simulating the dynamical oscillator model. C) Comparison of simulated islet activity as a function of percent excitable cells (Pexc) for cubic and sphere packing architectures in the dynamical model for physiological wild-type gap junction conductance. (TIF) [file pcbi.1003819.s005.tif]

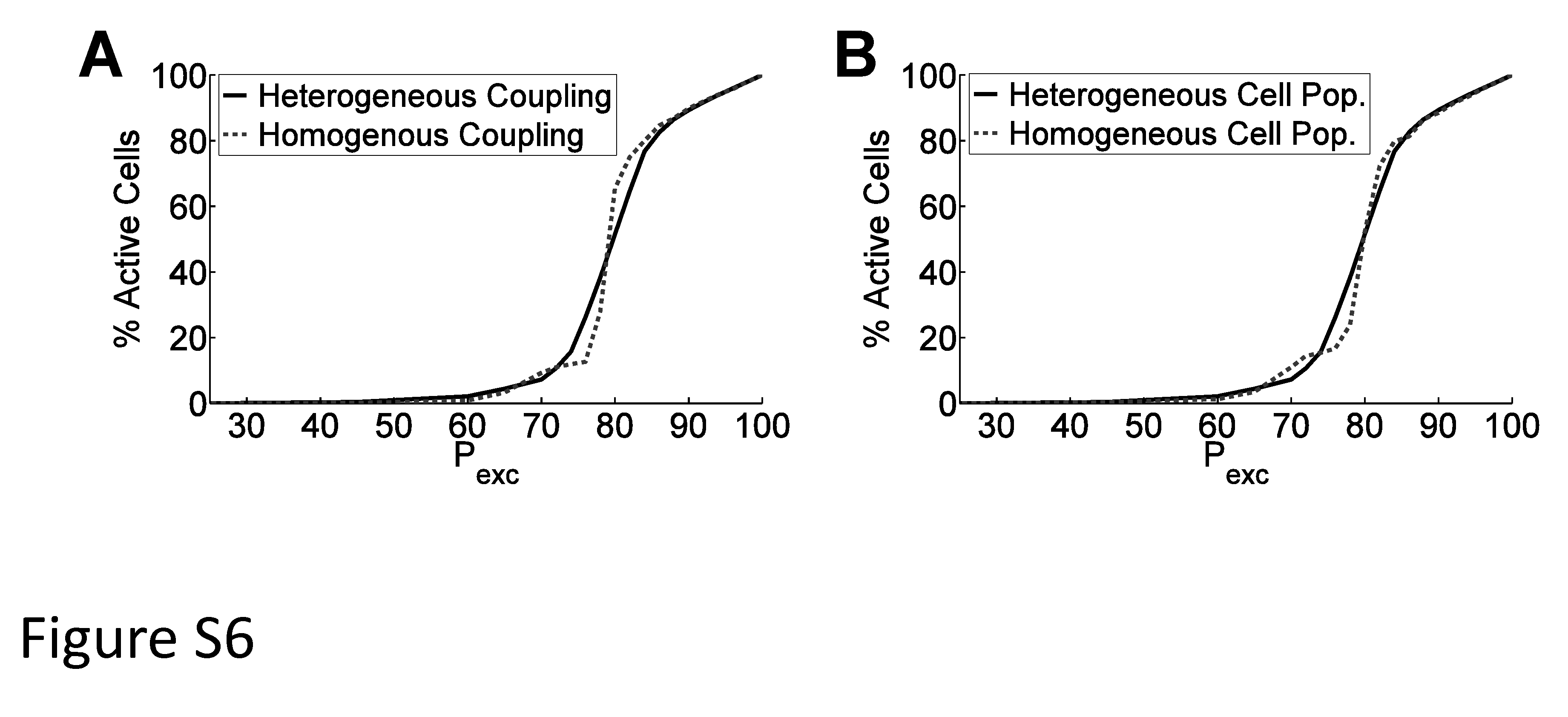

Supplement: Figure S6 — Effects of endogenous cellular heterogeneity and coupling heterogeneity on phase transition behavior. A) Simulated islet activity as a function of percent excitable cells (Pexc) in the presence and absence of heterogeneous distributions of gap junction coupling conductance in the dynamical model. B.) Simulated islet activity as a function of percent excitable cells (Pexc) in the presence and absence of heterogeneous distributions of cell physiology parameters in the dynamical model. (TIF) [file pcbi.1003819.s006.tif]

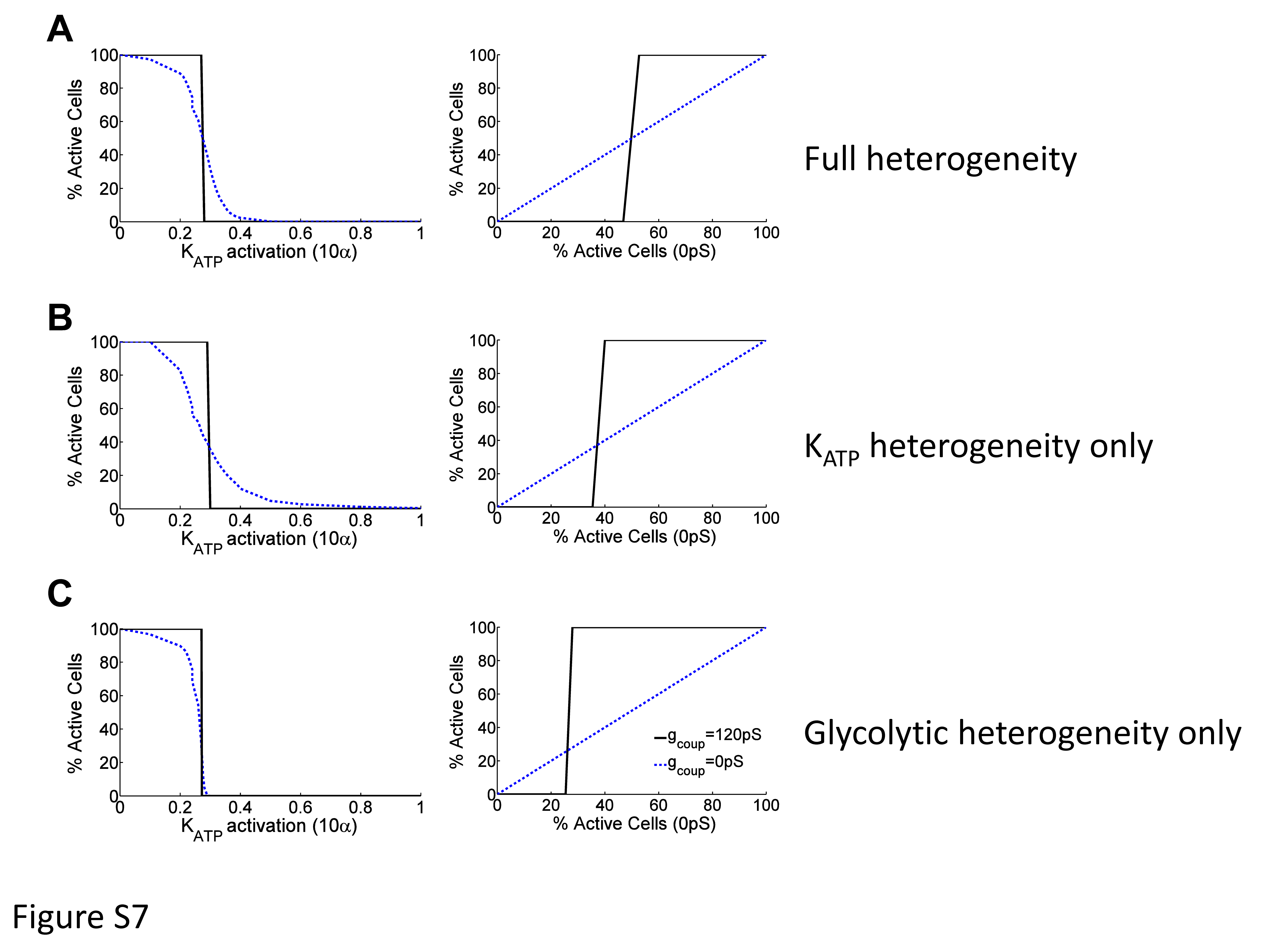

Supplement: Figure S7 — Dependence of phase transitions in β-cell network activity on the origin of endogenous β-cell heterogeneity in simulated islets. A) Left: Percent cells showing [Ca2+]i elevations in simulated islet as a function of a uniform increase in the fraction of ATP-insensitivity of KATP channel activation (α) across cells of the islet. Heterogeneity is present in all parameters described in Table S1 and as used elsewhere in this study. Mean simulation data is presented for zero gap junction conductance (0 pS) and wild-type gap junction conductance (120 pS). Right: activity of fully-coupled islet system as a function of activity in the uncoupled islet systems which represents the excitability of the constituent cells, with heterogeneity present in all parameters. For islets lacking gap junction coupling, with zero gap junction conductance, the result is trivially linear (dashed). B) As in A for heterogeneity solely in KATP channel activity. C) As in A for heterogeneity solely in glycolytic flux. (TIF) [file pcbi.1003819.s007.tif]

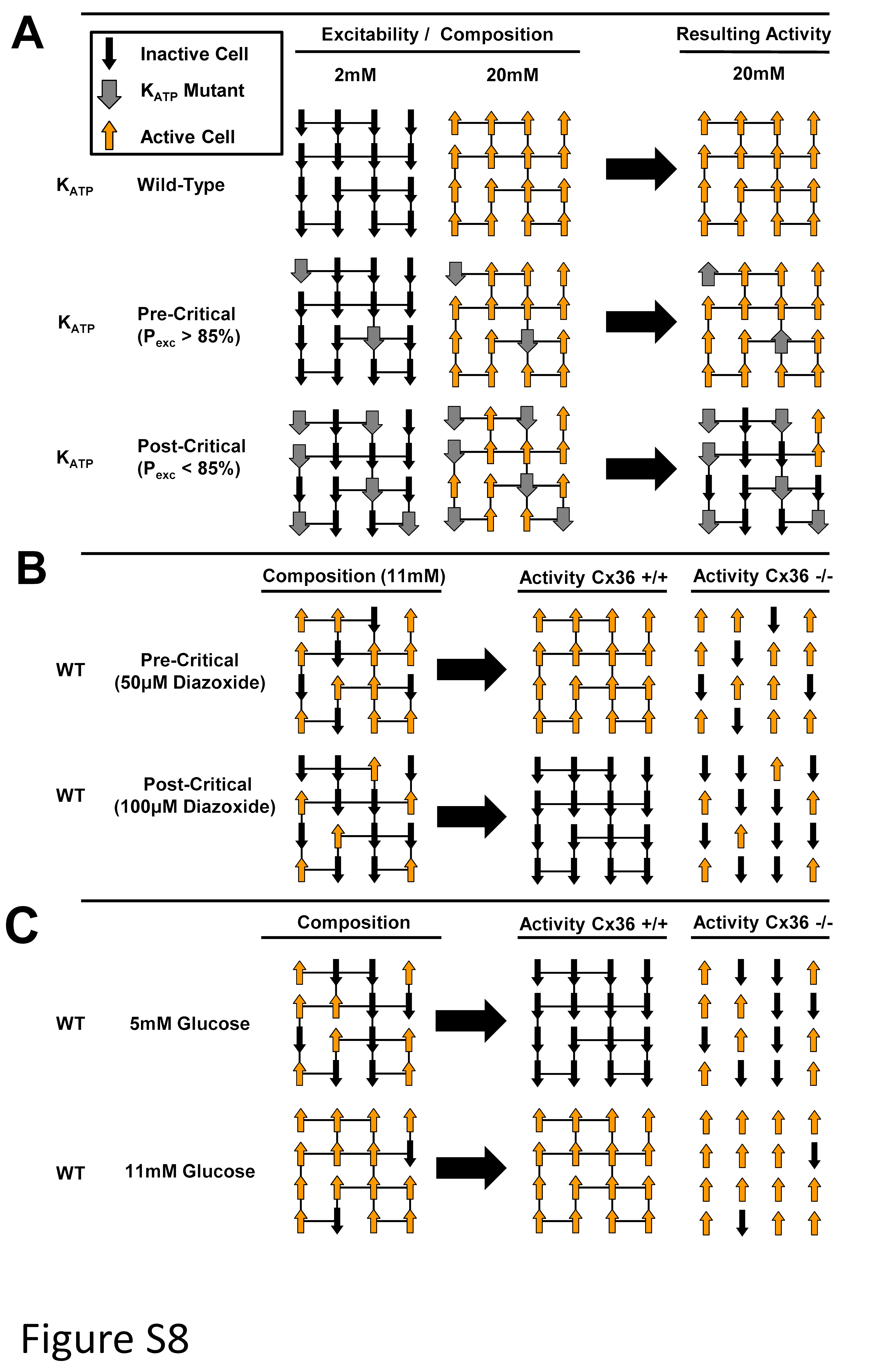

Supplement: Figure S8 — Mean-field theory analogy of β-cell network activity. The excitability of constituent units (i.e. their glucose sensitivities) and the resulting network activity takes into account coupling and different experimental perturbations. A) Cells of wild-type islets are inexcitable at low glucose (2 mM) and all excitable at high glucose (20 mM), therefore wild-type islets are respectively fully inactive and fully active. Cells expressing Kir6.2[ΔN30,K185Q] are glucose-insensitive and constitutively inexcitable. When Kir6.2[ΔN30,K185Q] penetrance is <15% (Pexc>0.85) there are insufficient inexcitable cells to suppress global activity, and so coupling leads to inexcitable cells being recruited to be active. However, when Kir6.2[ΔN30,K185Q] penetrance rises above ∼15% (Pexc<0.85), global quiescence ensues where the majority of normally excitable cells are rendered inactive. B) For low diazoxide (<50 µM), there are fewer excitable cells compared to untreated, however if coupling exists then all cells are recruited to be active. However, in the absence of coupling the resulting activity is the same as the composition, which results in lower activity. In the case of higher diazoxide treatments (>100 µM) the proportion of inexcitable cells exceeds the threshold for suppression. If coupling exists then all cells are rendered inactive. However in the absence of coupling the resulting activity is the same as the composition and some cells remain active. C) This mechanism can also explain how the islet maintains a robust well-defined glucose-stimulated response, but not in the absence of gap junction coupling. As glucose is increased more cells become excitable, but in the presence of coupling if the excitable fraction is less than a critical threshold (e.g. at ∼5 mM glucose), all cells are rendered inactive and the islet is quiescent. At a glucose level where the excitable fraction exceeds the critical threshold (e.g. ∼11 mM glucose) all cells are recruited to be active. However in [file pcbi.1003819.s008.tif]
